# Suppressing Mesenchymal Stromal Cell Ferroptosis Via Targeting a Metabolism‐Epigenetics Axis Corrects their Poor Retention and Insufficient Healing Benefits in the Injured Liver Milieu

**DOI:** 10.1002/advs.202206439

**Published:** 2023-02-19

**Authors:** Guangyu Hu, Zhe Cui, Xiyao Chen, Fangfang Sun, Tongzheng Li, Congye Li, Ling Zhang, Xiong Guo, Hang Zhao, Yunlong Xia, Wenjun Yan, Wei Yi, Miaomiao Fan, Rongjin Yang, Shan Wang, Ling Tao, Fuyang Zhang

**Affiliations:** ^1^ Department of Cardiology Xijing Hospital The Fourth Military Medical University Xi'an 710032 P. R. China; ^2^ Department of Geriatrics Xijing Hospital The Fourth Military Medical University Xi'an 710032 P. R. China; ^3^ Department of Cardiovascular Surgery Xijing Hospital The Fourth Military Medical University Xi'an 710032 P. R. China

**Keywords:** branched‐chain amino acid transaminase‐1, ferroptosis, liver injury, mesenchymal stromal cells, retention

## Abstract

Mesenchymal stromal cell (MSC) implantation is a promising option for liver repair, but their poor retention in the injured liver milieu critically blunts therapeutic effects. The aim is to clarify the mechanisms underlying massive MSC loss post‐implantation and establish corresponding improvement strategies. MSC loss primarily occurs within the initial hours after implantation into the injured liver milieu or under reactive oxygen species (ROS) stress. Surprisingly, ferroptosis is identified as the culprit for rapid depletion. In ferroptosis‐ or ROS‐provoking MSCs, branched‐chain amino acid transaminase‐1 (BCAT1) is dramatically decreased, and its downregulation renders MSC susceptible to ferroptosis via suppressing the transcription of glutathione peroxidase‐4 (GPX4), a vital ferroptosis defensing enzyme. BCAT1 downregulation impedes GPX4 transcription via a rapid‐responsive metabolism‐epigenetics coordinating mechanism, involving *α*‐ketoglutarate accumulation, histone 3 lysine 9 trimethylation loss, and early growth response protein‐1 upregulation. Approaches to suppress ferroptosis (e.g., incorporating ferroptosis inhibitors in injection solvent and overexpressing BCAT1) significantly improve MSC retention and liver‐protective effects post‐implantation. This study provides the first evidence indicating that excessive MSC ferroptosis is the nonnegligible culprit for their rapid depletion and insufficient therapeutic efficacy after implantation into the injured liver milieu. Strategies suppressing MSC ferroptosis are conducive to optimizing MSC‐based therapy.

## Graphical abstract

Here, ferroptosis is identified as the culprit for mesenchymal stromal cell (MSC) depletion post‐implantation into the injured liver milieu. A metabolism‐epigenetics axis involving BCAT1‐mediated BCAA metabolism and H3K9me3 controls MSC ferroptosis vulnerability via regulating GPX4 (a key ferroptosis‐defensing enzyme) transcription. Strategies inhibiting ferroptosis (incorporating ferroptosis inhibitors or overexpressing BCAT1) are feasible to improve MSC therapy.

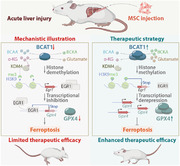

## 1 Introduction

Mesenchymal stromal/stem cells (MSCs) are a multipotent cell population frequently used in cellular therapy due to their unique advantages, including wide origins, tissue reparative potential, low immunogenicity, and low ethical concerns.^[^ ^1^ ^]^ Mounting evidence reveals that MSCs implanted into the acutely or chronically injured liver offer therapeutic benefits via a variety of mechanisms, e.g., attenuating hepatocyte death, modulating immune responses, reducing fibrotic processes, and releasing liver‐protective cytokines.^[^ ^2^, ^3^ ^]^ Supported by a series of basic and clinical investigations, MSC implantation has been recognized as a promising option for acute and chronic liver diseases and is gradually stepping into clinical practice.^[^ ^4^, ^5^ ^]^ Though visible progress has been achieved in this field, there remain unsolved weaknesses constraining the therapeutic effectiveness of MSCs. Among the limitations, poor MSC retention in the disadvantageous milieu is considered a primary cause of cell loss and insufficient therapeutic effectiveness after implantation into injured tissues.^[^ ^6^, ^7^ ^]^ Thus, feasible strategies to enhance MSC retention in the local microenvironment of the damaged liver are urgently required to optimize MSC‐based therapy.

A widely accepted theory is that the majority of MSCs end in death after implantation into the injured or diseased liver. When encountering pathological stresses beyond their maximal tolerance in the adverse milieu, MSCs will enter into an irreversible cell death program, rather than stably colonize and offer therapeutic effects.^[^ ^1^, ^8^ ^]^ Thus, promoting MSC survival at the implantation site is considered a critical way to increase their retention and improve therapeutic effectiveness. Since apoptosis was coined in the 1950s, a series of regulated cell death (RCD) forms, e.g., necroptosis, pyroptosis, ferroptosis, and lately, cuproptosis, have been discovered in the past decades.^[^ ^9^, ^10^ ^]^ Each form of RCD appears to have more or less evidence to support its prevalence in MSCs under pathological stress.^[^ ^11^, ^12^, ^13^ ^]^ So far, it remains unclear which form of RCD is the culprit for excessive MSC loss when delivered into the injured or diseased liver milieu, and because of this reason, the corresponding interventions are not yet established.

Since the conception of RCD, the different types of RCD have been inextricably linked with the burst of reactive oxygen species (ROS).^[^ ^14^ ^]^ ROS initiates, interacts with, and is decisive in a series of RCD via complex mechanisms, such as causing damage to cellular organelles and the plasma membrane.^[^ ^15^ ^]^ Moreover, the burst of ROS incurred by oxidative stress is a vital pathogenic factor usually seen in the local milieu of diseased livers, such as drug hepatotoxicity, hepatic ischemia/reperfusion injury, alcoholic or non‐alcoholic fatty liver disease, and cirrhosis.^[^ ^16^, ^17^ ^]^ In the present study, we observed that a rapid and tremendous loss of MSCs occurred in the initial hours upon in vitro ROS stress or after in vivo implantation into the injured liver induced by carbon tetrachloride (a hepatic toxin). This rapid loss in this early time window accounted for the majority of the total cell loss in the whole process. Based on this observation, the present study aimed to identify which form of RCD is responsible for MSC loss in the early phase after implantation into the injured liver or upon ROS stress. Furthermore, the underlying mechanisms and preclinical strategies to optimize MSC therapy are explored.

## 2 Results

### 2.1 Excessive Ferroptosis is the Main Culprit for the Rapid MSC Depletion after Implantation into the Injured Liver Milieu or Upon ROS Stress

Adipose‐derived MSCs (ADSCs) were isolated from healthy Sprague‐Dawley rats and were in vitro labeled with an enhanced green fluorescent protein (EGFP). These ADSCs were injected into the recipient rats, whose livers were injured by the toxin carbon tetrachloride (CCl_4_). ADSC retention was determined by EGFP fluorescent signals at a series of time points after implantation. Intriguingly, ≈60% of ADSCs were rapidly lost in the initial 6 h and 90% disappeared in the 24 h post‐implantation (Figure S1A, Supporting Information). A similar declining trend was seen via measuring EGFP mRNA levels (Figure S1B, Supporting Information). These results confirm that ADSCs are frustratingly intolerant to the injured liver milieu and undergo a rapid depletion post‐implantation.

**Figure 1:**
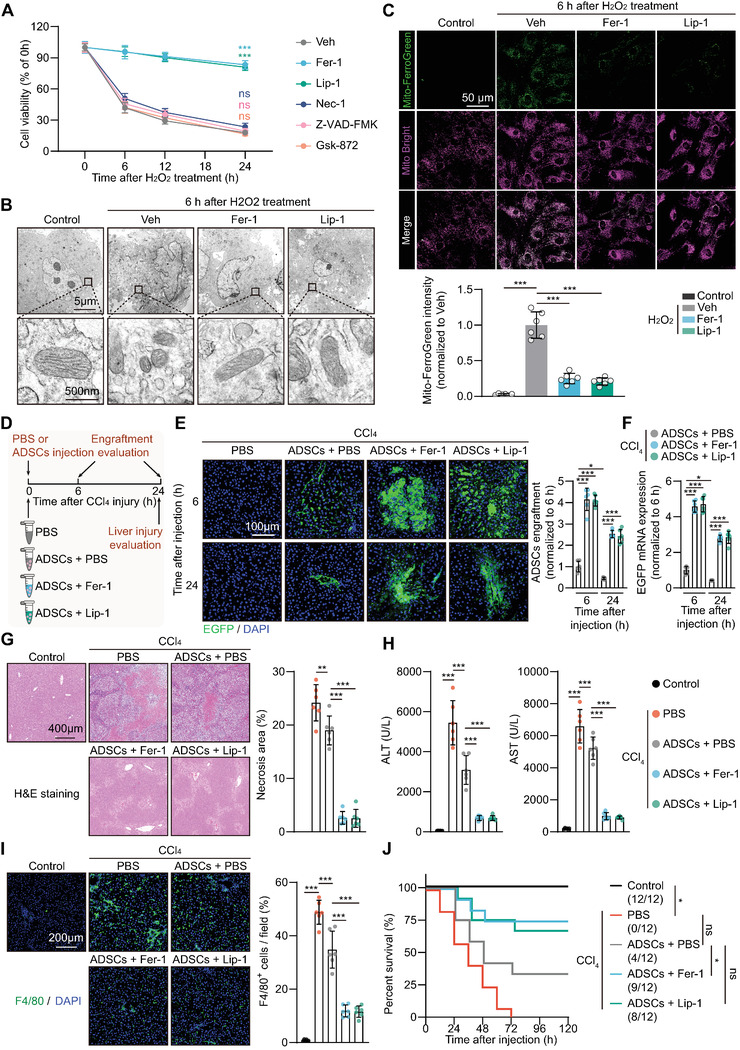
Excessive ferroptosis leads to rapid MSC depletion after implantation into the injured liver or upon ROS stress. A) Cell viability time course at a period of 24 h of ADSCs treated with 200 µM H_2_O_2_ in the presence of vehicle (Veh, DMSO), Fer‐1 (2 µM, ferroptosis inhibitor), Lip‐1 (2 µM, ferroptosis inhibitor), Z‐VAD‐FMK (50 µM, pan‐caspase inhibitor), Nec‐1 (10 µM, RIPK1 inhibitor), or Gsk‐872 (10 µM, RIPK3 inhibitor). Cell viability of each group at 0 h was normalized as 100%. Statistics were cells treated with indicated inhibitors compared to the Veh group 24 h (*n* = 6 biological replicates). B) Representative images of transmission electron microscope (TEM) analysis of ADSCs treated with DMSO (Control) or 200 µM H_2_O_2_ for 6 h in the presence of Veh, Fer‐1 (2 µM), or Lip‐1 (2 µM). C) Representative images (top) and corresponding quantification (bottom) of mitochondrial Fe^2+^ in ADSCs treated with DMSO (Control) or 200 µM H_2_O_2_ for 6 h in the presence of Veh, Fer‐1 (2 µM), or Lip‐1 (2 µM). Mitochondrial Fe^2+^ ions were labeled by the Mito‐FerroGreen (green), and the mitochondria were labeled by the MitoBright (deep red). The intensity of Mito‐FerroGreen in H_2_O_2_ + Veh group was normalized as 1 (*n* = 6 biological replicates). D) Illustration of animal models. The CCl_4_‐injured livers were treated with PBS, ADSCs + PBS, ADSCs + Fer‐1, or ADSCs + Lip‐1. Fer‐1 and Lip‐1 were dissolved in the PBS solvent at a final concentration of 2 µM. E,F) Engraftment evaluation of EGFP‐labeled ADSCs at 6 and 24 h post‐injection. Engraftment was evaluated by immunostaining of EGFP E) and RT‐qPCR of EGFP mRNA levels F). The CCl_4_ + PBS group was used as negative control. Engraftment at 6 h of ADSCs + PBS was normalized as 1 (*n* = 6 rats per group). G) Representative images of H&E staining of liver sections at 24 h post‐CCl_4_ injury (left). Quantification of Necrosis area (right) (*n* = 6 rats per group). H) Serum ALT and AST levels at 24 h post‐CCl_4_ injury (*n* = 6 rats per group). I) Immunostaining of F4/80 and quantification of F4/80^+^ cells of liver sections at 24 h post‐CCl_4_ injury (*n* = 6 rats per group). Percent means the proportions of F4/80‐positive cells to the total DAPI‐positive cells per field. J) Kaplan‐Meier survival curves of the animals (*n* = 12 rats per group). Data are presented as mean ± SD. Data shown in (A), (C), (G), (H), and (I) were analyzed by one‐way ANOVA followed by a Bonferroni post hoc test. Data shown in (E) and (F) were analyzed by two‐way ANOVA followed by a Bonferroni post hoc test. Data shown in (J) were analyzed by log‐rank Mantel‐Cox test. *p < 0.05, **p < 0.01, ***p < 0.001, and ns means not significant.

The burst of ROS in the injured liver milieu was in vitro simulated by adding hydrogen peroxide (H_2_O_2_) into the cultured ADSCs. In line with the in vivo observation, a tremendous ADSC loss, accounting for nearly 70% of the total cells, occurred in the first 6 h of ROS stress (**Figure** **1**A). In an attempt to determine which RCD participates in this process, a series of RCD inhibitors were separately added into the cells upon ROS challenge, including Z‐VAD‐FMK (a pan inhibitor of caspase, suppressing apoptosis and pyroptosis), necrostatin‐1 (Nec‐1, a receptor‐interacting protein kinase‐1 (RIPK1) inhibitor, suppressing necroptosis), Gsk‐872 (a receptor‐interacting protein kinase‐3 (RIPK3) inhibitor, suppressing necroptosis), ferrostatin‐1 (Fer‐1, an inhibitor of lipid peroxidation, suppressing ferroptosis), and liproxstatin‐1 (Lip‐1, an inhibitor of lipid peroxidation, suppressing ferroptosis). Surprisingly, among these RCD inhibitors, only Fer‐1 and Lip‐1 prevented ADSC death in the early phase upon ROS stress (Figure 1A). Through transmission electron microscopy, the shrunken mitochondria disappeared cristae, and ruptured mitochondrial membranes, together representing the ultrastructural characteristics of ferroptosis, were observed in ADSCs stressed by ROS for 6 h (Figure 1B). Aberrantly elevated iron ions in the mitochondrial is a hallmark of ferroptotic cells.^[^ ^18^ ^]^ Using the Mito‐FerroGreen, a fluorescent probe that detects ferrous ions (Fe^2+^) in the mitochondria, we found that Fe^2+^ was significantly accumulated in the mitochondria of ADSCs 6 h after ROS stress, indicating that ferroptosis occurs in ADSCs in the early stages of ROS‐provoked injury (Figure 1C). Intriguingly, these ferroptosis‐associated changes were significantly attenuated by Fer‐1 or Lip‐1 co‐treatment, providing direct evidence indicating that ferroptosis is the main culprit for ROS‐induced ADSC depletion.

We next delivered an equal number of ADSCs into the injured liver in the injection solvent separately supplemented with phosphate buffer saline (PBS), Fer‐1, or Lip‐1 (Figure 1D). At 3 h after injection, EGFP‐labeled ADSCs were isolated from the injured liver, and the expression levels of a number of ferroptosis‐associated marker genes, including Chac glutathione specific gamma‐glutamylcyclotransferase‐1 (*Chac1*), prostaglandin‐endoperoxide synthase‐2 (*Ptgs2*), solute carrier family‐7 member‐11 (*Slc7a11*), and acyl‐CoA synthetase long chain family member‐4 (*Acsl4*), were measured. We observed that the mRNA levels of *Chac1*, *Ptgs2*, *Slc7a11*, and *Acsl4* were significantly increased in ADSCs after delivery into the injured liver (Figure S2B, Supporting Information). A significant elevation in PTGS2 and SLC7A11 protein expression was further validated in the ADSCs isolated from the injured liver (Figure S2C, Supporting Information). When ADSCs were co‐treated with Fer‐1 or Lip‐1 in the cell solvent, all of the above molecular changes were greatly attenuated (Figure S2A,B, Supporting Information). These in vivo results indicate that the ADSCs were undergoing ferroptosis after delivering into the injured liver, which could be prevented by co‐treatment with the ferroptosis inhibitor. The cell‐free solvents with PBS, Fer‐1 or Lip did not influence CCl_4_‐induced liver injury, as evidenced by the unchanged serum alanine transaminase (ALT) and aspartate transaminase (AST) levels (Figure S3A,B, Supporting Information). This is perhaps due to the extremely low doses of chemical agents in the solvent for the recipient animals. However, in comparison to the ADSCs + PBS group, Fer‐1, and Lip‐1 in cell solvent both astoundingly improved ADSC retention at 6 and 24 h after delivery into the injured liver, as measured by EGFP fluorescent signals and mRNA levels (Figure 1E,F). The presence of Fer‐1 or Lip‐1 in cell solvent increased ADSC retention by 4 folds at 6 h and by 3 folds at 24 h post‐implantation (Figure 1E,F).

Consistent with the available evidence, ADSC implantation exerts an observable alleviation of CCl_4_‐induced liver injury, as evidenced by reduced liver necroptosis, less inflammatory cell infiltration, lower serum ALT and AST levels, and improved survival conditions (Figure 1G–J). Intriguingly, ADSCs supplemented with Fer‐1 or Lip‐1 in the injection solvent exhibited much superior therapeutic effects than those observed in the ADSCs + PBS group, as demonstrated by a significant decrease in liver necrotic lesions, less inflammatory cell infiltration, and lower serum ALT and AST levels (Figure 1H–J). Notably, in comparison to the ADSCs + PBS group, the survival rates of the animals challenged by CCl_4_ were significantly increased when receiving ADSCs + Fer‐1 or ADSCs + Lip‐1 treatment (Figure 1J). In contrast, the supplementation of the other RCD inhibitors in the cell solvent, including Z‐VAD‐FMK, Nec‐1, or Gsk‐872, did not show any improvement in their retention rate or therapeutic effectiveness when implanted into the injured liver (Figure S4A–E, Supporting Information).

To simulate the clinical application, we administered ADSCs to the rat liver 6 h after an acute CCl_4_ hit. The retention rate and therapeutic benefits of ADSCs on liver injury were found to be significantly improved by the addition of the ferroptosis inhibitor Fer‐1 or Lip‐1 in the cell solvent, as evidenced by increased ADSC implantation, lower serum ALT and AST levels, fewer liver necrotic lesions, less inflammatory cell infiltration, and a higher survival rate (Figure S5A–F, Supporting Information). In vitro preconditioning is a widely used strategy to mobilize endogenous adaptation or protection mechanisms to strengthen the MSC retention and healing effects.^[^ ^1^ ^]^ ADSCs were preconditioned with Fer‐1 or Lip‐1 for 24 h and then subjected to ROS or Erastin stress at different time points after Fer‐1 or Lip‐1 was withdrawn. The results showed that Fer‐1 or Lip‐1 preconditioning mediated a slight protective effect after 6 h of withdrawal but could not play a beneficial role in resisting ROS or Erastin damage after 12 h and 24 h of withdrawal (Figure S6A,B, Supporting Information). These results showed that, when the injury occurs, the protective effect of Fer‐1 and Lip‐1 requires that they exist in the cells and cannot be stably maintained in the MSCs by preconditioning. Taken together, for the first time, these in vitro and in vivo results show that excessive ferroptosis, probably not the other forms of RCD, is the primary culprit for the rapid and massive MSC loss following implantation into the injured liver milieu or in vitro ROS stress. More importantly, these findings imply that the main drawbacks of current MSC therapy, poor retention, and limited therapeutic effectiveness, can be addressed by suppressing MSC ferroptosis.

### 2.2 Identification of Branched‐Chain Amino Acid Transaminase‐1 (BCAT1) as a Critical Regulator of MSC Ferroptosis

**Figure 2:**
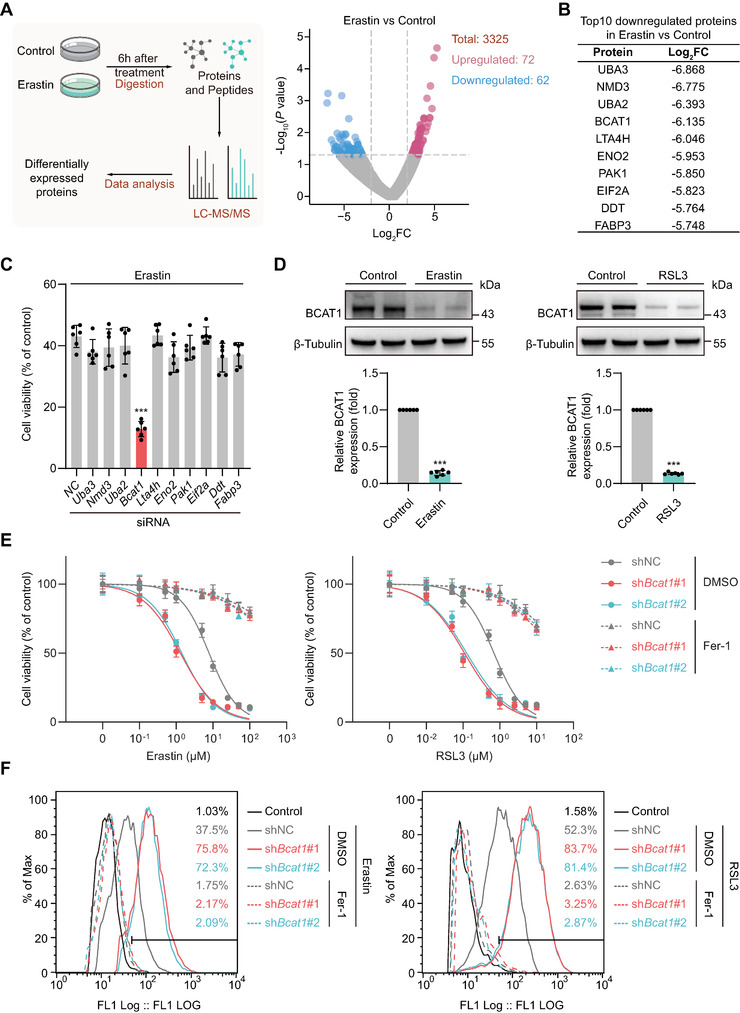
BCAT1 downregulation sensitizes MSCs to ferroptosis. A) ADSCs treated with DMSO (Control) or 10 µM Erastin for 6 h were subjected to shotgun proteomics. Left panel, scheme illustrating the workflow of shotgun proteomics. Right panel, the volcano plot showing the numbers of total identified proteins and differentially expressed proteins (DEPs) in the Erastin group versus Control identified by shotgun proteomics. The significantly upregulated or downregulated proteins are highlighted in red or blue, respectively. B) Ten most downregulated proteins upon Erastin treatment identified by shotgun proteomics. C) ADSCs were transfected with scrambled RNA (siNC) or siRNAs targeting *Uba3, Nmd3, Uba2, Bcat1, Lta4h, Eno2, Pak1, Eif2a, Ddt*, or *Fabp3*. 2 d after transfection, ADSCs were treated with DMSO (control) or Erastin (10 µM) for 6 h. Statistics were cells transfected with indicated siRNAs compared to cells transfected with siNC (*n* = 6 biological replicates). The experimental group that was given the same intervention but with the vehicle (DMSO) instead of Erastin was set as the control group, and the cell viability of the control group was set at 100%. D) Western blot analysis (top) and corresponding quantification (bottom) of BCAT1 expression in ADSCs treated with DMSO (Control), Erastin (10 µM), or RSL3 (1 µM) for 6 h. Control group was normalized as 1 (*n* = 6 biological replicates). E) ADSCs were transfected with shNC, sh*B*cat1#1, or sh*Bcat1*#2 upon indicated concentrations of Erastin (left) or RSL3 (right) treatment in the presence of DMSO or Fer‐1 (2 µM) for 6 h followed by the cell viability analysis (*n* = 6 biological replicates at each concentration in each group). F) ADSCs were transfected with shNC, sh*B*cat1#1, or sh*Bcat1*#2 upon Erastin (10 µM, left) or RSL3 (1 µM, right) treatment in the presence of DMSO or Fer‐1 (2 µM) for 6 h followed by lipid peroxidation analysis using the BODIPY 581/591 C11 staining (representative of 3 independent biological experiments). Data are presented as mean ± SD. Data shown in (C) were analyzed by one‐way ANOVA followed by a Bonferroni post hoc test. Data shown in (D) were analyzed by two‐tailed unpaired Student's t‐test. *p < 0.05, **p < 0.01, ***p < 0.001, and ns means not significant.

To explore the mechanism driving MSC ferroptosis and identify the potential intervention targets, the differentially expressed proteins (DEPs) in ADSCs treated by vehicle or Erastin (a ferroptosis inducer) for 6 h were analyzed by liquid chromatography‐mass spectrometry (LC‐MS)/mass spectrometry (MS) proteomics (**Figure** **2**A). Among 3325 proteins recognized, a total of 62 proteins were significantly decreased, whereas 72 proteins were upregulated, in the ADSCs undergoing ferroptosis (Figure 2A; Table S4, Supporting Information). Among the top ten downregulated proteins, small interfering RNA (siRNA) screening showed that BCAT1 silencing significantly exacerbated Erastin‐induced ADSC ferroptosis (Figure 2B,C). The enzyme BCAT1 catalyzes the first step of branched‐chain amino acids (BCAA) catabolism and transfers the amino group of BCAA to *α*‐ketoglutarate (*α*‐KG).^[^ ^19^ ^]^ The downregulation of BCAT1 was validated by Western blot in ferroptotic ADSC induced by Erastin or RSL3 treatment (Figure 2D). Next, BCAT1 expression was silenced in ADSCs via two independent short hairpin RNAs (shRNAs) (Figure S7A, Supporting Information). Under basal conditions, BCAT1 silencing did not influence ADSC survival and proliferation (Figure S7B, Supporting Information). Intriguingly, BCAT1 silencing significantly rendered ADSCs susceptible to ferroptosis in response to increasing doses of Erastin or RSL3 (Figure 2E). The production of lipid peroxidation, a core procedure of ferroptosis, was further increased in BCAT1‐silenced ADSC when ferroptosis was induced by Erastin or RSL3 (Figure 2F). The exacerbation of ferroptotic cell death and lipid peroxidation in BCAT1‐silencing ADSCs was fully prevented by Fer‐1 co‐treatment (Figure 2E, F). Autophagy mediated by the autophagy‐related protein 5 (ATG5) and ATG7 is reported to participate in ferroptosis by degrading ferritin.^[^ ^20^ ^]^ We asked whether these autophagic processes involve ferroptosis sensitivity modulated by BCAT1. ATG5 and ATG7 were separately silenced in ADSCs via the corresponding siRNA, and we found that even though ATG5 and ATG7 silencing moderately protected the ADSCs from ferroptosis induced by Erastin in the control ADSCs, the protective role of ATG5 and ATG7 silencing on ferroptosis disappeared in BCAT1‐silencing cells, suggesting that suppression of autophagy failed to rescue the ferroptosis vulnerability due to BCAT1 downregulation (Figure S8A,B, Supporting Information). Collectively, these results demonstrate that BCAT1 plays a critical but previously unrecognized role in the modulation of ADSC ferroptosis.

ADSCs were treated with H_2_O_2_ to simulate the ROS burst in the injured liver milieu. We observed that BCAT1 protein expression significantly decreased as early as 1 h upon ROS challenge and continued to decline over time until 6 h, indicating that BCAT1 expression is very sensitive to ROS stress (Figure S7C, Supporting Information). In comparison to the negative control group, ROS‐induced cell death and lipid peroxidation were markedly exacerbated in the BCAT1 silencing group (Figure S7D,E, Supporting Information). These adverse changes in BCAT1‐silenced ADSC were totally reversed by Fer‐1 or Lip‐1 co‐treatment (Figure S7D,E, Supporting Information). Collectively, these results demonstrate that BCAT1 is highly sensitive to ROS stress and its downregulation renders ADSC vulnerable to ROS‐associated ferroptosis.

### 2.3 Ferroptosis Vulnerability Induced by BCAT1 Downregulation Restricts MSC Retention and Therapeutic Efficacy in the Injured Liver

**Figure 3:**
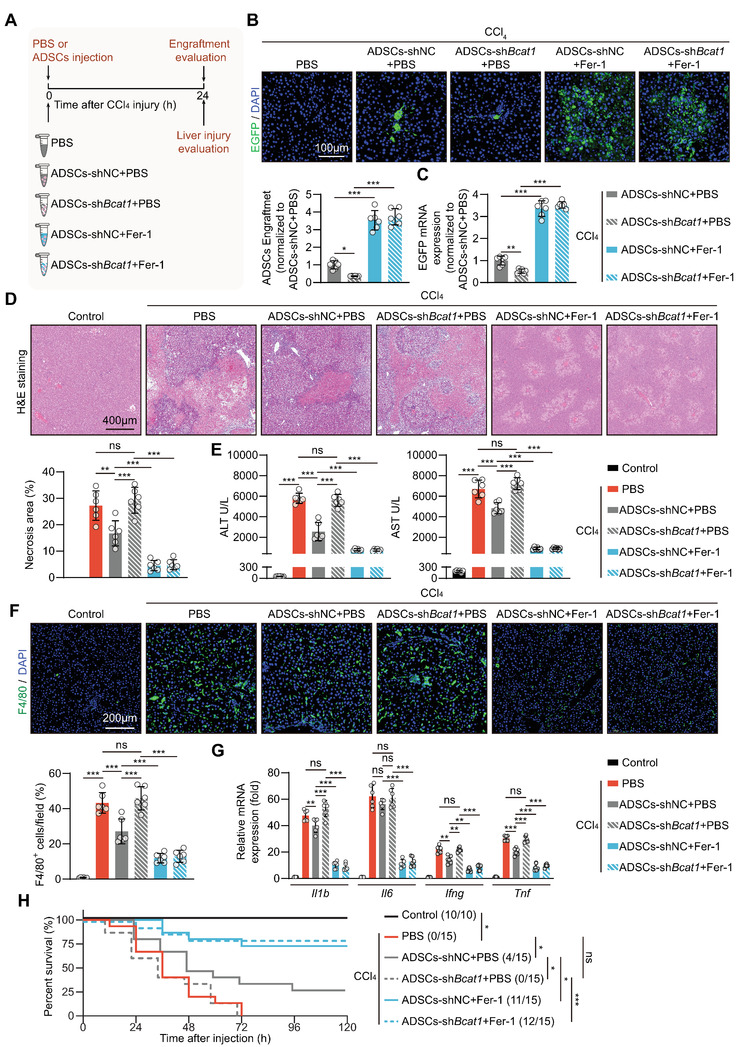
BCAT1 downregulation restricts MSC retention and therapeutic efficacy in the injured livers. A) Illustration of animal models. The CCl_4_‐injured livers were treated with either PBS alone or ADSCs + PBS, where ADSCs were transfected with shNC or sh*Bcat1* in combination with or without Fer‐1 (2 µM) dissolved in the PBS solvent. Engraftment of EGFP‐labeled ADSCs in livers at 24 h post‐injection, as evaluated by immunostaining of B) EGFP and C) RT‐qPCR of EGFP mRNA levels. The PBS group was used as negative control. The cell engraftment of the ADSCs‐shNC + PBS group was normalized as 1 (*n* = 6 rats per group). D) Representative images of H&E staining of liver sections at 24 h post‐CCl_4_ injury (top) and quantification of necrosis area (bottom) (*n* = 6 rats per group). E) Serum ALT and AST levels at 24 h post‐CCl_4_ injury (*n* = 6 rats per group). F) Immunostaining of F4/80 (top) and quantification of F4/80^+^ cells of liver sections at 24 h post‐CCl_4_ injury (bottom) (*n* = 6 rats per group). Percent means the proportions of F4/80‐positive cells to the total DAPI‐positive cells per field. G) mRNA expression of inflammatory cytokines *Il1b*, *Il6*, *Ifng*, and *Tnf* in livers as determined by RT‐qPCR at 24 h post‐CCl_4_ injury. The control group was normalized as 1 (*n* = 6 rats per group). H) Kaplan‐Meier survival curves of the animals (*n* = 10–15 rats per group). Data are presented as mean ± SD. Data shown in (B), (C), (D), (E), (F), and (G) were analyzed by one‐way ANOVA followed by a Bonferroni post hoc test. Data shown in (H) were analyzed by log‐rank Mantel‐Cox test. *p < 0.05, **p < 0.01, ***p < 0.001, and ns means not significant.

We next asked whether ferroptosis vulnerability provoked by BCAT1 downregulation affects MSC retention and healing effects when they were implanted into the injured liver. ADSCs transfected with negative control (ADSCs‐shNC) or BCAT1 shRNA (ADSCs‐sh*Bcat1*), co‐treated with vehicle or Fer‐1 in the solvent, were implanted into the injured liver (**Figure** **3**A). In comparison to the ADSCs‐shNC group, BCAT1 silencing significantly suppressed the retention of ADSCs in the injured liver milieu, as evidenced by reduced EGFP fluorescent signals and mRNA levels (Figure 3B,C). As a consequence, the alleviative effects of ADSCs on liver necroptosis, inflammatory cell infiltration, and proinflammatory cytokine production were almost abolished by BCAT1 silencing (Figure 3D–G). The improvement in the survival condition of animals that received ADSCs treatment was also eliminated when BCAT1 expression was silenced (Figure 3H). Suppressing MSC ferroptosis via Fer‐1 co‐treatment significantly improved the retention and liver‐protection efficacy in both control and BCAT1‐silenced ADSCs to the same levels (Figure 3D–H). Together, these results confirm that ferroptosis vulnerability induced by BCAT1 downregulation significantly restricts MSC retention and therapeutic efficacy in the injured liver.

### 2.4 BCAT1 Downregulation Suppresses the Ferroptosis Defense Enzyme Glutathione Peroxidase‐4 (GPX4) Expression via Increasing Intracellular *α*‐KG Levels

**Figure 4:**
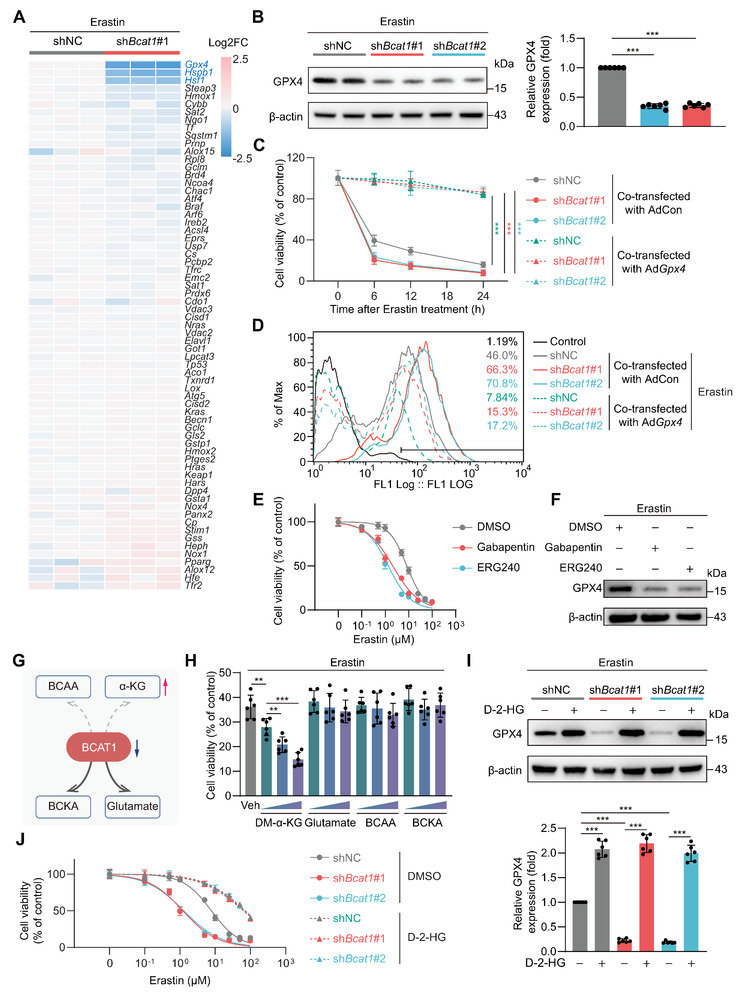
BCAT1 downregulation suppresses GPX4 expression via increasing intracellular *α*‐KG levels. A) ADSCs were transfected with shNC or sh*Bcat1*#1. 2 d after transfection, cells were challenged with Erastin (10 µM) for 6 h and subjected to the Ferroptosis qPCR array. Heatmap shows gene expression level detected by the Ferroptosis qPCR array. Gene expression levels were normalized as Log2(fold change in sh*Bcat1*#1 vs shNC). *Gpx4*, *Hspb1*, and *Hsf1* were found significantly downregulated in sh*Bcat1*#1 compared with shNC (*n* = 3 biological replicates). B) Western blot analysis (left) and corresponding quantification (right) of GPX4 protein expression in ADSCs transfected with shNC, sh*Bcat1*#1, or sh*Bcat1*#2 upon Erastin (10 µM, 6 h) treatment (*n* = 6 biological replicates). C) Cell viability time course at a period of 24 h of ADSCs upon Erastin (10 µM) treatment. ADSCs were transfected with shNC, sh*Bcat1*#1, or sh*Bcat1*#2 and co‐transfected with either control adenovirus vectors (AdCon) or GPX4 overexpression adenovirus vectors (Ad*Gpx4*). (*n* = 6 biological replicates). D) ADSCs were transfected with shNC, sh*Bcat1*#1, or sh*Bcat1*#2 and co‐transfected with either AdCon or Ad*Gpx4*. 2 d after transfection, cells were challenged with Erastin (10 µM) for 6 h and stained with the BODIPY 581/591 C11 for lipid peroxidation analysis (representative of 3 independent biological experiments). E) Cell viability of ADSCs upon increasing concentrations of Erastin in the presence of DMSO or the BCAT1 catalytic activity inhibitors, Gabapentin (10 mM) or ERG240 (10 mM) for 6 h (*n* = 6 biological replicates at each time point in each group). F) Western blot analysis of GPX4 protein expression in ADSCs challenged with Erastin (10 µM) in the presence of DMSO, Gabapentin (10 mM), or ERG240 (10 mM) for 6 h. G) Schematics of the catabolic reaction catalyzed by BCAT1. The downregulation of BCAT1 is supposed to result in intracellular *α*‐KG accumulation. H) Cell viability of ADSCs incubated with vehicle or increasing concentrations of DM‐*α*‐KG (1, 5, or 10 µM), Glutamate (0.1, 0.25, or 0.5 mM), BCAA (0.429, 1.716, or 3.432 mM), or BCKA (0.429, 1.716, or 3.432 mM) upon Erastin (10 µM) treatment for 6 h. Each metabolite and Erastin were added at the same time (*n* = 6 biological replicates). The experimental group that was given the same intervention but with the vehicle (DMSO) instead of Erastin was set as the control group, and the cell viability of the control group was set at 100%. I) Western blot analysis (top) and corresponding quantification (bottom) of GPX4 protein expression in ADSCs transfected with shNC, sh*Bcat1*#1, or sh*Bcat1*#2 upon Erastin (10 µM) treatment in combination with or without D‐2‐HG (5 µM) for 6 h (*n* = 6 biological replicates). J) Cell viability of ADSCs transfected with shNC, sh*Bcat1*#1, or sh*Bcat1*#2 upon increasing concentrations of Erastin treatment in combination with either DMSO or D‐2‐HG (5 µM) for 6 h (*n* = 6 biological replicates). Data are presented as mean ± SD. Data shown in (B), (C), and (H) were analyzed by one‐way ANOVA followed by a Bonferroni post hoc test. Data shown in (I) were analyzed by two‐way ANOVA followed by a Bonferroni post hoc test. *p < 0.05, **p < 0.01, and ***p < 0.001.

We next explored the mechanisms underlying BCAT1 regulation of MSC ferroptosis. We compared the differentially expressed genes (DEGs) in control or BCAT1 shRNA‐transfected ADSCs upon Erastin stimulation via a real‐time PCR assay detecting ferroptosis‐related genes. As shown in the heatmap, the mRNA levels of Gpx4, Hspb1, and Hsf1 were significantly decreased, whereas none was significantly upregulated, in BCAT1‐silenced ADSCs (**Figure** **4**A). The downregulation of GPX4 protein, rather than HSPB1 or HSF1, was further observed in BCAT1‐silenced ADSCs upon Erastin treatment (Figure 4B; Figure S9A, Supporting Information). GPX4 is a master defender of ferroptosis.^[^ ^21^ ^]^ To determine whether GPX4 involves in BCAT1‐mediated modulation of ferroptosis, we overexpressed wild‐type GPX4 in ADSCs via adenovirus‐mediated gene transfer (Figure S9B, Supporting Information). Restoration of GPX4 expression in BCAT1‐silenced ADSCs totally rescued their vulnerability to Erastin‐induced ferroptosis and lipid peroxidation (Figure 4C,D).

BCAT1 is a metabolic enzyme catalyzing the first step of BCAA catabolism. We next asked whether BCAT1 modulates ferroptosis sensitivity through its enzymatic activity. We added two individual BCAT1 activity inhibitors, gabapentin, and ERG240, into cultured ADSCs. Inhibition of BCAT1 enzymatic activity via gabapentin or ERG240 significantly exacerbated ADSC ferroptosis in response to increasing concentrations of Erastin (Figure 4E). Similar to the observation in BCAT1‐silenced ADSCs, gabapentin and ERG240 markedly induced GPX4 downregulation in ASDCs upon Erastin stimulation (Figure 4F). These results reveal that the enzymatic activity of BCAT1 is indispensable for its regulation of ferroptosis.

BCAT1 transfers the amino groups from BCAA to *α*‐KG and generates glutamate and the corresponding branched‐chain alpha‐keto acids (BCKA) (Figure 4G). As expected, BCAT1 silencing significantly increased intracellular *α*‐KG and BCAA levels, whereas it decreased BCKA and glutamate concentrations (Figure S9C, Supporting Information). We next supplemented BCAA, BCKA, glutamate, and dimethyl‐*α*‐KG (DM‐*α*‐KG, a cell‐permeable derivative of *α*‐KG) at different concentrations into the cultured ADSC upon Erastin challenge. Among these metabolites, only DM‐*α*‐KG increased Erastin‐induced ferroptosis in a dose‐dependent manner, indicating that *α*‐KG might be a key metabolite involved in BCAT1‐mediated ferroptosis modulation (Figure 4H). *α*‐KG is a necessary metabolite that fuels the tricarboxylic acid (TCA) cycle and is also required for dioxygenase activation as a co‐factor.^[^ ^22^ ^]^ In BCAT1‐silenced ADSCs, intracellular levels of TCA cycle metabolites, including citrate, succinate, fumarate, and malate, remained unchanged (Figure S9D, Supporting Information). We thus added disodium (R)‐2‐Hydroxyglutarate (D‐2‐HG), a competitive antagonist of *α*‐KG‐dependent dioxygenases, into BCAT1‐silenced ADSCs. Intriguingly, we observed that D‐2‐HG rescued the downregulation of GPX4 and the exacerbation of ferroptosis in BCAT1‐silenced ADSCs upon Erastin challenge (Figure 4I,J). These in vitro results demonstrate that BCAT1 regulates GPX4 expression and ferroptosis sensitivity in MSCs primarily through its enzymatic activity‐mediated *α*‐KG metabolism.

### 2.5 BCAT1 Regulates GPX4 Transcription via a Metabolism‐Epigenetics Coordinating Mechanism

**Figure 5:**
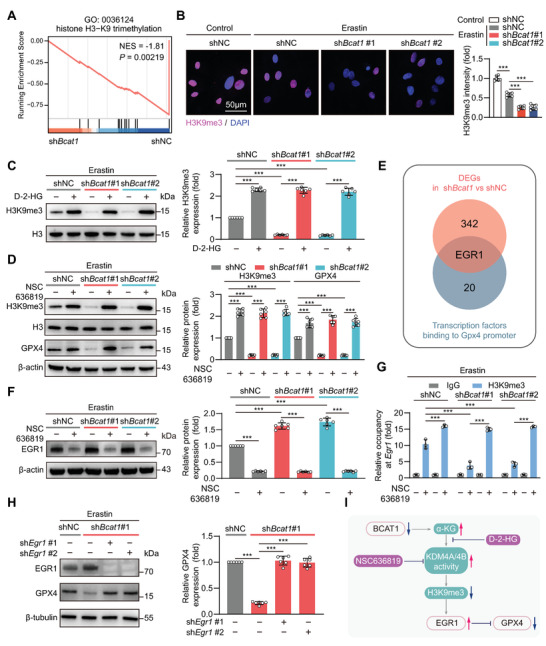
BCAT1 regulates GPX4 transcription via a metabolism‐epigenetics coordinating mechanism. A) GSEA showing the enrichment of H3K9me3 (GO: 0036124). ADSCs were transfected with shNC or sh*Bcat1*#1. 2 d after transfection, cells were challenged with Erastin (10 µM) for 6 h followed by the RNA‐seq. B) Immunostaining of H3K9me3 (red) in ADSCs transfected with shNC, sh*Bcat1*#1, or sh*Bcat1*#2. 2 d after transfection, cells were challenged with Erastin (10 µM) for 6 h followed by the immunostaining analysis. ADSCs transfected with shNC at basal conditions without Erastin treatment were used as control, the intensity of which was normalized as 1 (*n* = 6 biological replicates). C) Western blot analysis (left) and corresponding quantification (right) of H3K9me3 protein expression in ADSCs transfected with shNC, sh*Bcat1*#1, or sh*Bcat1*#2 in combination with or without D‐2‐HG (10 µM) upon Erastin (10 µM) treatment for 6 h (*n* = 6 biological replicates). D) Western blot analysis (left) and corresponding quantification (right) of H3K9me3 and GPX4 protein expression in ADSCs transfected with shNC, sh*Bcat1*#1, or sh*Bcat1*#2 in combination with or without the KDM4A/B inhibitor NSC 636819 (5 µM) upon Erastin (10 µM) treatment for 6 h (*n* = 6 biological replicates). E) Venn diagram showing the number of DEGs (342, top) and transcription factors (20, bottom). DEGs were identified in RNA‐seq of ADSCs transfected with shNC or sh*Bcat1*#1 under the following threshold: absolute fold change > 1.5 and *p*‐value < 0.05. Transcription factors binding to *Gpx4* promoter region were obtained using the CistromeDB toolkit with RP score > 0.80. The overlapping region recognizes EGR1 only. F) Western blot analysis (left) and corresponding quantification (right) of EGR1 protein expression in ADSCs transfected with shNC, sh*Bcat1*#1, or sh*Bcat1*#2 in combination with or without NSC 636819 (5 µM) upon Erastin (10 µM) treatment for 6 h (*n* = 6biological replicates). G) H3K9me3 occupancy at *Egr1* promoter region in ADSCs transfected with shNC, sh*Bcat1*#1, or sh*Bcat1*#2 in combination with or without KDM4A/B inhibitor NSC 636819 (5 µM) upon Erastin (10 µM) treatment for 6 h as determined by ChIP‐qPCR (*n* = 3 biological replicates). H) Western blot analysis (left) and corresponding quantification (right) of EGR1 and GPX4 protein expression in ADSCs transfected with shNC or sh*Bcat1*#1 and co‐transfected with shNC, sh*Egr1*#1, or sh*Egr1*#2 upon Erastin (10 µM) treatment for 6 h (*n* = 6biological replicates). I) Schematic illustration of the cellular mechanisms. Data shown in (B) and H) were analyzed by one‐way ANOVA followed by a Bonferroni post hoc test. Data shown in (C), (D), (F), and (G) were analyzed by two‐way ANOVA followed by a Bonferroni post hoc test. *p < 0.05, **p < 0.01, and ***p < 0.001.

*α*‐KG regulates epigenetic homeostasis, e.g., DNA and histone methylation, as a co‐factor of dioxygenases.^[^ ^23^ ^]^ Given that D‐2‐HG rescued GPX4 downregulation and ferroptosis vulnerability in BCAT1‐silenced ADSCs, we hypothesized that the epigenetic changes regulated by *α*‐KG‐dependent dioxygenases underlie this process. We analyzed the RNA‐seq data obtained from control or BCAT1‐silenced ADSCs upon Erastin stress. It is notable that histone 3 lysine 9 trimethylation (H3K9me3) represented the most significantly changed epigenetic pathways between negative control and BCAT1‐silenced ADSC (**Figure** **5**A). H3K9me3 abundance was significantly decreased in ADSCs during Erastin‐induced ferroptosis and these changes were exacerbated in BCAT1‐silenced cells, as measured by fluorescent staining and Western blot (Figure 5B and Figure S10A, Supporting Information). D‐2‐HG significantly suppressed ferroptosis‐related H3K9me3 loss in BCAT1‐silenced ADSCs to the same levels as control, indicating the indispensable role of *α*‐KG in regulating H3K9me3 abundance (Figure 5C). Lysine demethylases 4A and 4B (KDM4A/4B) are the *α*‐KG‐dependent demethylases specific for H3K9me3 in mammalian cells.^[^ ^24^ ^]^ We observed that KDM4A/4B inhibition by their specific inhibitor NSC 636819 markedly rescued the H3K9me3 loss and GPX4 downregulation in both control and BCAT1‐silenced ADSCs in the existence of Erastin (Figure 5D). These results demonstrate that the loss of H3K9me3 mediated by *α*‐KG‐dependent KDM4A/4B is an essential step for GPX4 downregulation and ferroptosis vulnerability in BCAT1‐silenced MSCs.

H3K9me3 is a histone modification suppressing gene transcription, yet the occupancy of H3K9me3 in the *Gpx4* promoter region remained unchanged in BCAT1‐silenced ADSCs when compared to the control group (Figure S10B, Supporting Information). This evidence suggests GPX4 downregulation is not directly attributed to H3K9me3 loss in BCAT1‐silenced ADSCs. We thus aimed to identify the transcriptional factor (TF) directly responsible for GPX4 downregulation in BCAT1‐silenced ADSCs. The RNA‐seq data reveals that, among the candidate TFs regulating *Gpx4* transcription, the early growth response protein‐1 (EGR1) was the unique molecule with significant changes in BCAT1‐silenced ADSCs (Figure 5E). A conserved EGR1 binding motif was confirmed in the *Gpx4* promoter region (Figure S10C, Supporting Information). EGR1 expression was significantly upregulated in ADSCs undergoing Erastin‐induced ferroptosis, which was further increased in BCAT1‐silenced cells. Suppression of H3K9me3 loss by NSC 636819 significantly blocked ERG1 upregulation in both control and BCAT1‐silenced ADSC to the same level upon Erastin stress (Figure 5F). The chromatin immunoprecipitation (ChIP) assay confirmed the occupancy of H3K9me3 in the *Egr1* promoter region was reduced in BCAT1‐silenced ADSCs when compared to the control, which was fully prevented by the co‐treatment of KDM4A/4B inhibitor NSC 636819 (Figure 5G). These observations suggest that the loss of H3K9me3 directly contributes to ERG1 upregulation in BCAT1‐silenced ADSCs. In ADSCs transfected with a plasmid carrying EGR1, Gpx4 mRNA levels and promoter transcription activity was significantly suppressed in a dose‐dependent manner (Figure S10D,E, Supporting Information), confirming that EGR1 is a direct TF suppressing the transcription of Gpx4. Intriguingly, EGR1 knockdown significantly rescued GPX4 downregulation in BCAT1‐silenced ADSCs in the presence of Erastin (Figure 5H). The ferroptosis susceptibility of BCAT1‐silenced ADSCs was rescued by EGR1 knockdown (Figure S10F, Supporting Information). These results reveal a novel metabolism‐epigenetics coordinating mechanism, involving *α*‐KG accumulation, H3K9me3 loss, and EGR1 upregulation, which underlies BCAT1 downregulation‐mediated GPX4 transcriptional suppression and ferroptosis vulnerability (Figure 5I).

### 2.6 BCAT1 Overexpression Increases GPX4‐Dependent Ferroptosis Resistance and Improves MSC Retention and Therapeutic Efficacy

After clarifying that BCAT1 downregulation renders MSCs vulnerable to ferroptosis, we thus asked whether BCAT1 overexpression increases the ferroptosis resistance of MSCs and improves their retention and healing effects post‐implantation. BCAT1 overexpression was achieved by adenovirus‐mediated gene transfer in ADSCs (Figure S11A, Supporting Information). BCAT1 overexpression significantly preserved GPX4 expression in cultured ADSCs and protected the cells against ferroptosis upon Erastin challenge (Figure S11B,D, Supporting Information). Notably, GPX4 silencing via shRNA transfection significantly abolished the anti‐ferroptosis effect mediated by BCAT1 overexpression (Figure S11C,D, Supporting Information). These in vitro results demonstrate that BCAT1 overexpression increases ferroptosis resistance in MSCs via the GPX4‐dependent manner.

**Figure 6:**
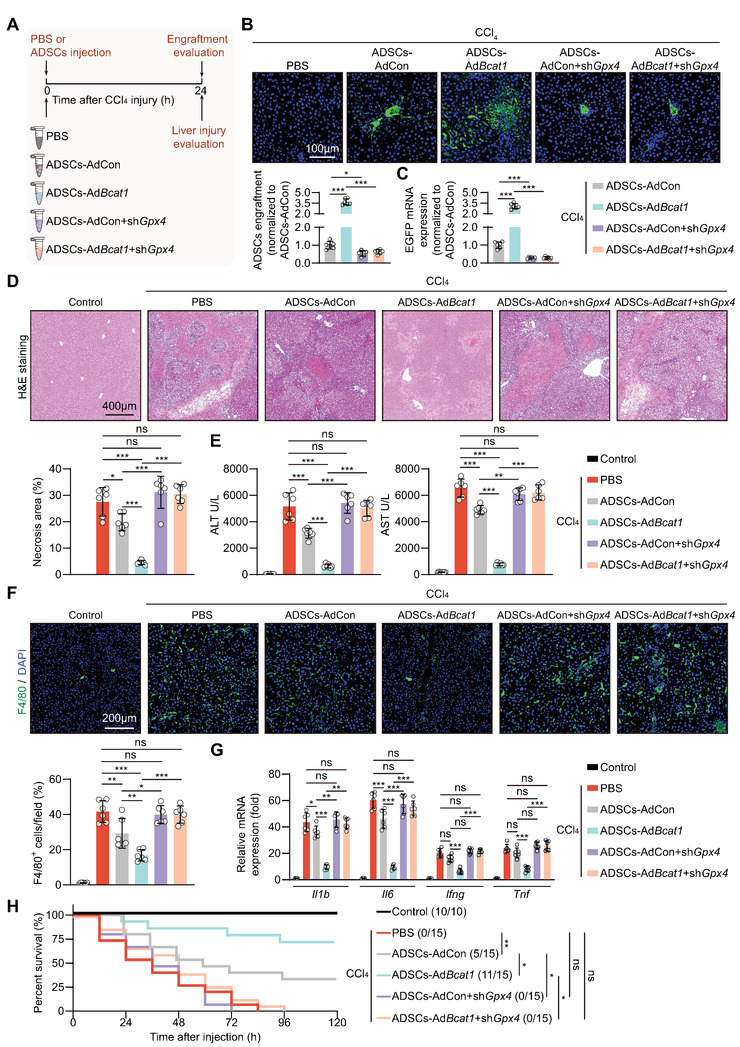
BCAT1 overexpression increases MSC retention and therapeutic efficacy in a GPX4‐dependent manner. A) Illustration of animal models. The CCl_4_‐injured livers were treated with either PBS alone or ADSCs + PBS, where ADSCs were transfected with AdCon or Ad*Bcat1*, with or without sh*Gpx4* co‐transfection. Engraftment of EGFP‐labeled ADSCs in livers at 24 h post‐injection, as evaluated by immunostaining of B) EGFP and C) RT‐qPCR of EGFP mRNA levels. The PBS group was used as negative control. The cell engraftment of the ADSCs‐AdCon group was normalized as 1 (*n* = 6 rats per group). D) Representative images of H&E staining of liver sections at 24 h after liver injury (top) and quantification of necrosis area (bottom) (*n* = 6 rats per group). E) Serum ALT & AST levels at 24 h after liver injury (*n* = 6 rats per group). F) Representative immunostaining images of F4/80 (top) and quantification of F4/80^+^ cells of liver sections at 24 h post liver injury (bottom) (*n* = 6 rats per group). Percent means the proportions of F4/80‐positive cells to the total DAPI‐positive cells per field. G) mRNA expression of inflammatory cytokines *Il1b*, *Il6*, *Ifng*, and *Tnf* in livers at 24 h post‐injury as determined by RT‐qPCR. The Control group was normalized as 1 (*n* = 6 rats per group). H) Kaplan‐Meier survival curves of the animals (*n* = 10–15 rats per group). Data are presented as mean ± SD. Data shown in (B), (C), (D), (E), (F), and (G) were analyzed by one‐way ANOVA followed by a Bonferroni post hoc test. Data shown in (H) were analyzed by log‐rank Mantel‐Cox test. *p < 0.05, **p < 0.01, ***p < 0.001, and ns means not significant.

Next, control (ADSCs‐AdCon) or BCAT1‐overexpressed (ADSCs‐AdBcat1) ADSCs, transfected with or without Gpx4 shRNA, were locally delivered into the injured livers of rats challenged by CCl_4_ (**Figure** **6**A). In comparison to the ADSCs‐AdCon group, BCAT1 overexpression significantly increased the retention in the injured liver milieu, as evidenced by increased EGFP fluorescent signals and mRNA levels (Figure 6B). In line with the increasing retention rates, ADSCs‐AdBcat1 exhibited much superior therapeutic effects than those of ADSCs‐AdCon after implantation into the injured liver, as evidenced by fewer necroptosis lesions, lower serum ALT and AST levels, less inflammatory cell infiltration, and lower proinflammatory cytokine generation (Figure 6C–G). The survival rate of the animals that received ADSCs‐AdBcat1 treatment was much higher than that of the rats treated by ADSCs‐AdCon (Figure 6H). Notably, the above improvements in the retention and liver‐protective effects were completely abolished in both ADSCs‐AdCon and ADSCs‐AdBcat1 cells when their ferroptosis resistance was erased via GPX4 knockdown (Figure 6B–H). These results demonstrate that BCAT1 overexpression is a feasible strategy to improve MSC retention and therapeutic efficacy via increasing GPX4‐dependent resistance to ferroptosis.

### 2.7 Suppressing Ferroptosis by Incorporating a Ferroptosis Inhibitor in Cell Solvent or Overexpressing BCAT1 Improves Human MSC Retention and Liver‐Protection

**Figure 7:**
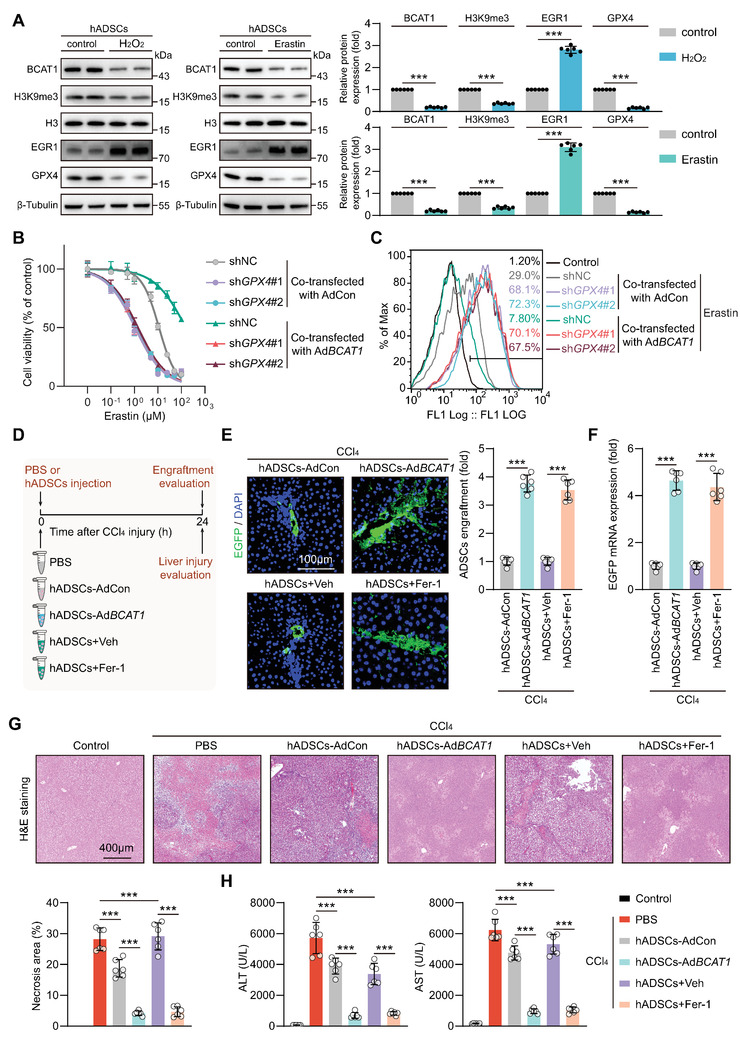
BCAT1 overexpression or ferroptosis inhibition enhances human MSC retention and therapeutic efficacy in the injured livers. A) Human ADSCs (hADSCs) were challenged with DMSO (Control), H_2_O_2_ (200 µM), or Erastin (10 µM) for 6 h, followed by Western blot analysis of BCAT1, H3K9me3, EGR1, and GPX4 protein expression (*n* = 6 biological replicates). B) Cell viability of hADSCs transfected with shNC, sh*Gpx4*#1, or sh*Gpx4*#2 and co‐transfected with either AdCon or Ad*Bcat1* upon increasing concentrations of Erastin treatment for 6 h (*n* = 6 biological replicates). C) hADSCs were transfected with shNC, sh*Gpx4*#1, or sh*Gpx4*#2 and co‐transfected with either AdCon or Ad*Bcat1* upon Erastin (10 µM) treatment for 6 h followed by lipid peroxidation analysis using BODIPY 581/591 C11 staining (representative of 3 independent biological experiments). D) Illustration of animal models. The CCl_4_‐injured livers were treated with either PBS alone or hADSCs + PBS. The hADSCs were transfected with AdCon or Ad*Bcat1*, in combination with Veh (vehicle) or Fer‐1 (2 µM) dissolved in the PBS solvent. Engraftment of EGFP labeled hADSCs in livers at 24 h post‐injection, as evaluated by immunostaining of E) EGFP and F) RT‐qPCR of EGFP mRNA levels. The PBS group was used as negative control. The cell engraftment of the hADSCs‐AdCon group was normalized as 1 (*n* = 6 rats per group). G) Representative images of H&E staining of liver sections at 24 h after liver injury (top) and quantification of necrosis area (bottom) (*n* = 6 rats per group). H) Serum ALT and AST levels at 24 h after liver injury (*n* = 6 rats per group). Data are presented as mean ± SD. Data shown in (A), (E), and (F) were analyzed by two‐tailed unpaired Student's *t*‐test. Data shown in (B), (G), and (H) were analyzed by one‐way ANOVA followed by a Bonferroni post hoc test. *p < 0.05, **p < 0.01, and ***p < 0.001.

To test the clinical translational potential of the strategies to suppress ferroptosis, we observed the molecular changes in human ADSCs (hADSCs) challenged by H2O2 or Erastin, separately. Like the findings in rat ADSCs, H2O2 or Erastin exposure resulted in similar molecular changes in hADSCs, including BCAT1 downregulation, H3K9me3 loss, ERG1 upregulation, and GPX4 suppression (**Figure** **7**A). BCAT1 was overexpressed via adenovirus‐mediated gene transfer (hADSCs‐AdBCAT1) and GPX4 expression was knocked down by shRNA transfection in hADSCs (Figure S12A,B, Supporting Information). In comparison to hADSCs‐AdCon, Erastin‐induced ferroptosis, and ferroptosis‐related lipid peroxidation were significantly attenuated in hADSCs‐AdBCAT1 (Figure 7B,C). These anti‐ferroptosis effects mediated by BCAT1 overexpression were greatly weakened when GPX4 was silenced (Figure 7B,C), confirming that BCAT1 overexpression protects hADSCs against ferroptosis via increasing GPX4‐dependent ferroptosis defense. Strategies suppressing ferroptosis via incorporating Fer‐1 into the injection solvent or overexpressing BCAT1 in hADSCs were evaluated in rats suffering from CCl_4_‐induced liver injury (Figure 7D). Suppressing hADSCs ferroptosis significantly increased their retention after implantation into the injured liver milieu, as measured by EGFP fluorescent signals and mRNA levels (Figure 7E,F). Moreover, strategies to inhibit hADSCs ferroptosis improved the hepatoprotective effects as evidenced by fewer necroptotic lesions and lower serum ALT and AST levels (Figure 7G,H). These in vivo and in vitro data provide evidence indicating that strategies suppressing human MSC ferroptosis (e.g., incorporating ferroptosis inhibitors in the solvent or overexpressing BCAT1) tackle their poor retention in the injured milieu and improve their therapeutic effectiveness.

## 3 Discussion

Effective prevention or treatment of liver injury is a fundamental issue in the management of hepatic diseases. MSCs implanted into an injured or diseased liver provide therapeutic benefits via a variety of mechanisms, making them one of the most promising strategies for relieving hepatocyte damage in both acute and chronic liver diseases.^[^ ^3^, ^25^ ^]^ Insufficient MSC retention, on the other hand, causes a rapid depletion of MSCs and severely limits their therapeutic efficacy due to their intolerance of the unfavorable milieu of the injured liver.^[^ ^26^ ^]^ Suppressing MSC death and enhancing their preservation in the local milieu are thus considered an unneglected direction to optimize MSC‐based therapy. This issue, unfortunately, remains elusive and the corresponding interventions are currently lacking.

Aiming to address the aforementioned issue, we have made a series of novel and significant findings in the present study. First, the most important finding of this study is that we identify ferroptosis as a predominant cause of MSC loss in the early phase after implantation into the injured liver or ROS stress. When delivered into the injured liver, MSCs are rapidly and massively lost in the initial 6 h. This loss accounts for a majority of the total cell depletion after implantation. This early, rapid, and extensive loss of MSCs is also observed in vitro when stressed by ROS, the main pathogenic factor universally seen in acute and chronic liver diseases. These results emphasize that the early phase after implantation is a time window that cannot be missed to modulate MSC survival and preservation in the local milieu of the liver. At this early stage, MSCs exhibited the typical structural changes of ferroptosis. The ferroptosis inhibitors Lip‐1 and Fer‐1, rather than inhibitors of the other RCD forms, rescue the tremendous loss of MSCs, increase their retention, and improve their therapeutic efficacy in the injured liver. A similar phenomenon is also observed in MSCs exposed to in vitro ROS stress. Ferroptosis is a form of RCD driven by iron‐dependent lipid peroxidation.^[^ ^27^ ^]^ The burst of ROS has been well recognized as the most important stimulus for initiating and driving ferroptosis.^[^ ^18^ ^]^ Here we observe that, when implanted into the injured liver characterized by ROS burst (in vivo) or directly receiving ROS stress (in vitro), MSCs massively undergo ferroptotic death. These results for the first time identify ferroptosis as a prime culprit for MSC loss in the early phase and emphasize that targeted suppression of MSC ferroptosis is a feasible and effective approach to attenuate their depletion and increase their retention when implanted into the injured liver. It should be noted that while the concentration of ferroptosis inhibitor we added to the cell solvent was much lower than the pharmacological dose in vivo, this study cannot completely rule out the local influence of ferroptosis inhibitors in promoting tumorigenesis and drug resistance, given that ferroptosis plays an important role in the regulation of carcinogenesis and therapy resistance in acute and chronic liver diseases.^[^ ^28^ ^]^

Second, we identify BCAT1 as a critical molecule regulating the ferroptosis susceptibility of MSCs, especially under the circumstances of ROS stress. Even though the ferroptotic processes have been gradually clarified, the rheostat molecules controlling ferroptosis sensitivity or flux remain elusive. BCAT1 is an enzyme catalyzing the first step of BCAA catabolism.^[^ ^19^ ^]^ Using non‐bias proteomics, we surprisingly observe that BCAT1 expression is very responsive to ROS burst and ferroptotic stimuli, and its downregulation renders MSCs vulnerable to ROS or reagent‐induced ferroptosis. This observation identifies BCAT1 as a key molecule regulating the ferroptosis resistance of MSCs upon ROS stress. Importantly, BCAT1 overexpressed MSCs exhibit an increasing resistance or tolerance to ferroptosis when implanted into the injured liver or challenged by ROS. No matter if derived from rats or humans, MSCs with BCAT1 overexpression have a better retention situation and show superior therapeutic effectiveness in the injured liver. These results emphasize that suppressing ferroptosis via targeting BCAT1 is a feasible and promising strategy to optimize MSC‐based therapy.

Third, a novel mechanism coordinating metabolism and epigenetics in regulating GPX4 transcription and ferroptosis susceptibility has been established. As the only enzyme capable of reducing phospholipid hydroperoxides, GPX4 is a critical molecule that protects cells from ferroptosis.^[^ ^21^ ^]^ Through a series of experiments, we identify GPX4 as an indispensable molecule responsible for BCAT1's regulation of ferroptosis. Notably, BCAT1 enzymatic activity is required for GPX4 transcription in MSCs, implying that the metabolites BCAA, BCKA, glutamate, or *α*‐KG are involved in this process.^[^ ^29^ ^]^ BCAT1 downregulation results in an intracellular accumulation of *α*‐KG in MSCs, as seen in a variety of cancer cells.^[^ ^30^, ^31^ ^]^ *α*‐KG is a metabolite directly entering the TCA cycle or indirectly modulating histone or DNA methylation through the dioxygenases.^[^ ^23^ ^]^ Based on bioinformatics‐guided experiments, we clarify that BCAT1 downregulation results in *α*‐KG accumulation increases *α*‐KG‐dependent KDM4A/B activity, and suppresses H3K9me3 modification. H3K9me3 is a histone medication that suppresses gene expression, and its loss is linked to MSC aging and injury.^[^ ^32^ ^]^ Here, we observe that the loss of H3K9me3 in EGR1 promoter loci causes a rapid upregulation of EGR1, a member of the early responsive TFs to stress.^[^ ^33^ ^]^ EGR1 directly binds to the GPX4 promoter region, suppresses GPX4 transcription, and thus sensitizes MSCs to ferroptosis. Notably, the downregulation of BCAT1 occurs in the initial 1 to 3 h after ROS stress, suggesting that this mechanism is very responsive to ROS or ferroptosis stress and thus underlies the ferroptotic cell loss in the early phase upon implantation into the injured liver milieu. These results establish a novel and critical mechanism regulating GPX4 transcription and ferroptosis resistance via coordinating the BCAA‐*α*‐KG metabolism and H3K9me3 epigenetic changes in the cells.

Finally, we confirm the effectiveness of the strategies targeting ferroptosis in human MSCs. Human MSCs, including ADSCs, umbilical cord MSCs (UCMSCs), or bone marrow MSCs (BMMSCs), are frequently used in treating acute or chronic liver diseases.^[^ ^3^, ^34^ ^]^ Taking hADSCs (the most commonly used cell types) as an example, we have tested the strategies targeting ferroptosis in animal models with severe liver injury. Suppressing ferroptosis of hADSCs via co‐treatment with ferroptosis inhibitors or overexpressing BCAT1 significantly increases their retention and improves the therapeutic efficacy in rat models suffering from CCl_4_‐induced liver injury. These results emphasize the translational prospective of the strategies targeting ferroptosis for the optimization of human MSC‐based therapy in treating a variety of diseases, such as liver, heart, and lung injuries.

There are some limitations to the present study. First, we only used the acute liver injury model. We did not check the models with chronic liver diseases such as cirrhosis or liver fibrosis, which call for more investigations. Second, in order to accurately evaluate MSC retention or tolerance in the injured liver microenvironment, we only used intrahepatic MSC delivery instead of the most commonly used clinical intravascular injection (e.g., hepatic artery, portal, or peripheral vein). Further research into the impact of MSC delivery fashion is required.

In summary, this study provides the first evidence demonstrating that ferroptosis is the main culprit for the rapid MSC depletion after implantation into the injured milieu or upon ROS stress. A novel metabolism‐epigenetics coordinating mechanism governed by BCAT1 is revealed in regulating ferroptosis susceptibility of MSCs. From the clinical translational perspective, strategies suppressing MSC ferroptosis are conducive to optimizing the retention and therapeutic effectiveness of MSCs.

## 4 Experimental Section

### ADSC Isolation and Culture

Adult male Sprague‐Dawley (SD) rats were used for the isolation of adipose‐derived mesenchymal stromal cells (ADSCs) as previously described.^[^ ^35^ ^]^ ADSCs were cultured in high‐glucose (HG) DMEM medium, supplemented with 10% fetal bovine serum and 1% streptomycin/penicillin. Passage 2 ADSCs were used in all experiments.

### Animal Models

All animal experiments in the current study were approved by the Animal Care and Use Committee of the Fourth Military Medical University (FMMU‐09‐122) and in accordance with the National Institutes of Health Guidelines for the Use of Laboratory Animals. To establish acute liver injury models, adult male SD rats, weighing 200–250 g, were anesthetized through inhalation of 2% isoflurane and were intraperitoneally injected with a single dose of 0.5 ml carbon tetrachloride (CCl_4_), dissolved in corn oil (1:1, vol/vol), per 100 g body weight.^[^ ^26^ ^]^ For animals receiving ADSCs or PBS treatment, right after CCl_4_ injection or 6 h after CCl_4_ injection, an incision (≈1.5 cm) on the upper right abdominal of skin and muscle was made to expose the liver, and a purse suture was placed over the incision. 2 × 10^6^ ADSCs suspended in 200 µL ice‐cold PBS, supplemented with or without ferroptosis inhibitors, were evenly administered into three different sites of the liver.^[^ ^36^ ^]^ After injection, the incision was closed using the previously placed purse suture. Animals treated only with corn oil were used as control. For liver injury analysis, the animals were sacrificed 24 h after CCl_4_ injection. For survival analysis, after CCl_4_ injection, the animals were observed every 12 h for a total period of 120 h. For the evaluation of ADSC engraftment, animals were sacrificed at indicated time points in the corresponding figure legends.

### ADSC Engraftment Evaluation

Adenoviruses carrying plasmids overexpressing enhanced green fluorescent protein (EGFP) were transfected into ADSCs for the evaluation of engraftment. For ADSCs with gene interference, cells were first transfected with EGFP, then passaged for further gene interference. The number of ADSCs engrafted to the liver was determined via EGFP immunostaining and quantitative polymerase chain reaction assessments of EGFP mRNA levels as previously described.^[^ ^37^ ^]^ For the quantification of EGFP‐labeled ADSC retention, liver tissue samples containing all three ADSC injection sites were collected, and the EGFP signals were detected by immunostaining. All the EGFP‐positive signals were counted using the ImageJ 1.53a software. The average values of the EGFP‐positive cell numbers in the three injection sites were calculated and represented as the ADSC engraftment level in the rat. six rats per group were analyzed. The images representing the average levels of the statistical results were shown in the corresponding figures. The following primary antibody was used for EGFP immunostaining: Anti‐EGFP Rabbit pAb (1:200, Servicebio, GB11602). The primers detecting EGFP mRNA are listed as follows: Forward (5′‐3′), CTGGTCGAGCTGGACGGCGACG‐3′; Reverse (5′‐3′), 5′‐CACGAACTCCAGCAGGACCATG.

### Measurement of Alanine Aminotransferase (ALT) and Aspartate Aminotransferase (AST)

Twenty‐four hours after CCl_4_ injection, animals were sacrificed, and blood serum was collected. The serum levels of AST and ALT were detected using a fully automated biochemical analyzer (Rayto, Shenzhen, China, Chemray 800). Enzyme activities were expressed in international units (U/L).

### Hematoxylin and Eosin (H&E) Staining and Analysis

Twenty‐four hours after CCl_4_ injection, liver samples containing the three ADSC injection sites were collected and fixed with 4% paraformaldehyde. H&E staining was performed as described before.^[^ ^38^ ^]^ The necrosis area of each sample was analyzed using ImageJ 1.53a software. The average values of the necrosis areas in three independent liver tissue samples were calculated and represented as the liver necrosis level in the rat. Six rats per group were analyzed. The images representing the average levels of the statistical results of each group were presented in the corresponding figures.

### Immunofluorescence Staining and Analysis

Tissue or cells were immunostained and analyzed as previously described.^[^ ^35^ ^]^ ImageJ 1.53a software was used for analysis. For the evaluation of F4/80^+^ cells, images from six rats per group were analyzed, and the images representing the mean values of statistical analysis in each group were presented in the corresponding figures. Primary antibodies used in current studies were listed as follows: anti‐EGFP (1:200, Servicebio, GB11602), anti‐F4/80 (1:200, Servicebio, GB11027), and anti‐H3K9me3 (1:200, ABclonal, A2360).

### ROS Treatment, Cell Death Inhibitors, and Drugs

ROS were generated by 200 µM hydrogen peroxide (Sigma‐Aldrich, H1009). Cell death inhibitors were used at the following concentrations: ferrostatin‐1 (Fer‐1, 2 µM TargetMol, T6500), liproxstatin‐1 (Lip‐1, 2 µM TargetMol, T2376), necrostatin‐1 (Nec‐1, 10 µM, TargetMol, T1847), Gsk‐872 (10 µM, TargetMol, T4074), Z‐VAD‐FMK (50 µM, TargetMol, T7020). 1S,3R‐RSL3 (TargetMol, T3646) and Erastin (TargetMol, T1765) were used as ferroptosis inducers. For KDM4A/KDM4B inhibition, cells were incubated with NSC 636819 (5 µM, TOCRIS, 5287). For BCAT1 activity inhibition, cells were treated with ERG240 (10 mM, Selleck, E1017) or gabapentin (10 mM, TargetMol, T0702). For BCAA incubation, cells were treated with 0.429, 1.716, or 3.432 mM BCAA mixture (weight ratios of valine: leucine: isoleucine = 1: 2: 1) as previously described.^[^ ^35^ ^]^ For BCKA incubation, cells were treated with 0.429, 1.716, or 3.432 mM BCKA mixture (weight ratios of *α*‐KIV: *α*‐KIC: *α*‐KMV = 1: 2: 1) as previously described.^[^ ^39^ ^]^ For *α*‐KG incubation, cells were treated with 1, 5, or 10 mM Dimethyl *α*‐ketoglutarate (Sigma‐Aldrich, 349631) in Figure 4H and 5 mM in further experiments. For glutamate incubation, cells were treated with 0.1, 0.25, or 0.5 mM l‐glutamic acid (Sigma‐Aldrich, G1251). For D‐2‐HG incubation, cells were treated with 5 mM disodium (R)‐2‐hydroxyglutarate (Sigma‐Aldrich, 16859).

### Transmission Electron Microscope (TEM)

ADSCs were treated with DMSO (Control) or 200 µM H_2_O_2_ for 6 h in the presence of vehicle, Fer‐1 (2 µM), or Lip‐1 (2 µM). After treatment, cells were fixed, cut into sections, and examined using a transmission electron microscope (JEM‐1400 Flash).^[^ ^27^ ^]^

### Mitochondrial Ferrous Ion Detection

The Mito‐FerroGreen (M489, Dojindo) fluorescent probe was utilized to assess mitochondrial ferrous ions (Fe^2+^) in live ADSCs.^[^ ^40^ ^]^ 2 × 10^3^ ADSCs were seeded on glass bottom culture dishes (801001, NEST) and incubated overnight. The next day, ADSCs were incubated with 5 µM Mito‐FerroGreen and co‐incubated with 100 nM the mitochondrial probe MitoBright LT Deep Red (MT12, Dojindo) for 30 min at 37 °C. After incubation, cells were washed with PBS twice and subjected to a serum‐free medium supplemented with DMSO (Control) or 200 µM H_2_O_2_ for 6 h in the presence of vehicle, Fer‐1 (2 µM), or Lip‐1 (2 µM). After treatment, cells were washed with PBS twice and observed using a confocal microscope (LSM880, Carl Zeiss). The intensity of Mito‐FerroGreen signal in each group was analyzed using ImageJ 1.53a software.

### Sorting of EGFP‐labeled ADSCs from CCl4‐Injured Livers

Rats receiving CCl_4_ were treated with EGFP‐labeled ADSCs supplemented with PBS, Fer‐1, or Lip‐1 in the solvent, separately. Fer‐1 and Lip‐1 were dissolved in 200 µL PBS solvent at a final concentration of 2 µM. 3 h post‐injection, animals were sacrificed under anesthesia and the liver samples near the ADSC injection sites were collected. The liver samples were cut into pieces, rinsed with ice‐cold PBS, dissociated using a tissue dissociation solution (abs9482, absin) for 15 min at 37 °C, and filtered through a 70 µm sterile filter. The cell suspension was then added with red cell lysis buffer and centrifuged at 1200 rpm for 5 min. After removal of supernatants, cells were resuspended and subjected to fluorescence‐activated cell sorting (FACS) using a MoFlo XDP cell sorter (Beckman Coulter) for the sorting of EGFP‐labeled ADSCs.

### Lipid Peroxidation Measurement

Lipid peroxidation was measured using BODIPY 581/591 C11 (Invitrogen, D3861). 2 × 105 ADSCs per well were seeded in 6‐well plates and incubated at 37 °C overnight. The next day, cells were treated with indicated drugs for 6 h, followed by incubation with fresh DMEM containing 5 µM BODIPY for 30 min at 37 °C. Cells were then harvested by trypsinization, washed twice with PBS, and resuspended in 200 µL PBS. Lipid peroxidation was assessed using an Epics XL‐MCL flow cytometer (Beckman Coulter) with a 488 nm laser on an FL1 detector. Flow cytometry data were analyzed using FlowJo v10 software (BD Biosciences).

### Cell Viability Measurements

Cell counting kit‐8 (cck‐8, C0005, TargetMol) was used for the measurement of cell viability as previously described.^[^ ^35^ ^]^ 2 × 10^3^ ADSCs were seeded in 96‐well plates per well. After treatment of drugs, cells were incubated with10 µL cck‐8 per well () at 37 °C for 2 h. The optical density (OD) was measured at wavelength of 450 nm using an Epoch Microplate Spectrophotometer (BioTek Instruments). The following formula was used to calculate cell viability (%): OD_treated_/OD_control_ × 100%, where control groups are ADSCs incubated with vehicle (PBS or DMSO) only without H_2_O_2_, Erastin, or Rsl‐3 challenges.

### Cell Proliferation Assay

For the evaluation of cell proliferation, CCK‐8 was used as described before.^[^ ^37^ ^]^ 1 × 10^3^ ADSCs were seeded in 96‐well plates per well. The day seeded cells adhered to plastic was determined as d0. The OD of cells were measured for four consecutive days. Cells incubated with CCK‐8 were changed to fresh medium each day after the OD was measured. The OD of d0 was normalized as 1, and the OD values of the following days were normalized to d0.

### Shotgun Proteomics

ADSCs were treated with 10 µM Erastin or vehicle or for 6 h. After treatment, cells were subjected to Shotgun proteomics, carried out by Applied Protein Technology Co. Ltd (Shanghai, China). In brief, proteins were extracted from cells, separated by SDS‐PAGE gel, and digested into peptides. The digested peptides were desalted on C18 Cartridges (Empore Extraction Disk Cartridge, Sigma‐Aldrich), concentrated by vacuum centrifugation, and reconstituted in formic acid.LC‐MS/MS analysis on a Q Exactive mass spectrometer coupled to Easy nLC (Thermo Fisher Scientific) was used for detection of peptides. MaxQuant software was used for identification and quantification analysis. Significance B was used for differential analysis and defined as a *p*‐value.^[^ ^41^ ^]^ Proteins under a threshold of absolute log2FC > 1 and *p* < 0.05 were considered as differentially expressed proteins (DEPs). Detailed information on proteins identified in shotgun proteomics was listed in Table S4 (Supporting Information).

### Western Blotting

Western blotting was carried out as described before.^[^ ^35^ ^]^ Image Lab 4.0 software (Bio‐Rad) was used for the analysis of blots. Primary antibodies used for western blotting were listed as follows: anti‐BCAT1 (1:1000, Proteintech, 13640‐1‐AP), anti‐*β*‐Tubulin (1:2000, Immunoway, YT4780), anti‐GPX4 (1:1000, ABclonal, A11243), anti‐*β*‐actin (1:2000, Immunoway, YM3028), anti‐H3K9me3 (1:2000, ABclonal, A2360), anti‐Histone H3 (1: 2000, ABclonal, A2348), anti‐EGR1 (1:2000, ABclonal, A2722), anti‐HSPB1 (1:1000, ABclonal, A16332), anti‐HSF1 (1:1000, ABclonal, A13765), anti‐FLAG (1:2000, ABclonal, AE005), anti‐PTGS2 (1:1000, ABclonal, A1253), anti‐SLC7A11 (1:1000, ABclonal, A2413), anti‐ATG5 (1:1000, ABclonal, A19677), and anti‐ATG7 (1:1000, ABclonal, A19604).

### Real‐Time Quantitative PCR (RT‐qPCR)

Total RNA was extracted from liver tissue or ADSCs using TRIzol reagent (Invitrogen, 15596026). RT‐qPCR was performed as previously described.^[^ ^35^ ^]^ Gene expression was calculated as 2^−∆∆Ct^ relative to *Actb* as the endogenous control. Detailed primers used for RT‐qPCR were listed in Table S1 in Supporting Information.

### Measurement of Intracellular Metabolite Concentration

ADSCs (2 × 10^6^ in quantity) transfected with shNC or sh*Bcat1*#1 were used for intracellular metabolite concentration measurement. Each metabolite level was determined using the following assay kits: BCAA detection kit (BioVision, K564‐100, *α*‐ketoglutarate assay kit (Sigma‐Aldrich, MAK054), glutamate assay kit (Sigma‐Aldrich, MAK004), citrate assay kit (Sigma‐Aldrich, MAK057), fumarate assay kit (Sigma‐Aldrich, MAK060), succinate assay kit (Sigma‐Aldrich, MAK184), and malate assay kit (Sigma‐Aldrich, MAK196). BCKA concentration was measured by high‐performance liquid chromatography (HPLC) as previously described.^[^ ^42^ ^]^ Intracellular metabolite levels were normalized to the ADSCs transfected with shNC.

### ADSC Transfection

Cells were transfected as previously described.^[^ ^35^ ^]^ Passage 2 ADSCs were used for transfection. Adenovirus vectors carrying empty plasmids (AdCon) or plasmids overexpressing rat or human BCAT1 (Ad*Bcat1*) and plasmids overexpressing rat GPX4 (Ad*Gpx4*) were purchased from Hanbio Co., Ltd. (China). Adenovirus carrying scrambled short hairpin RNA (shNC) or short hairpin RNAs targeting rat *Bcat1* (sh*Bcat1*#1 and sh*Bcat1*#2), rat *Egr1* (sh*Egr1*#1 and sh*Egr1*#2), rat *Gpx4* (sh*Gpx4*), and human *GPX4* (sh*GPX4*#1 and sh*GPX4*#2) were constructed by Hanbio Co., Ltd. as well. shRNAs targeting indicated genes are independent sequences. ADSCs at 60% confluence were transfected with indicated adenoviruses at an MOI of 50. 48 h after transfection, cells were used for future experiments.

Small interfering RNAs (siRNAs) targeting *Uba3*, *Nmd3 Uba2*, *Bcat1*, *Lta4h*, *Eno2*, *Pak1*, *Eif2a*, *Dbt*, *Fabp3*, *Atg5*, *Atg7*, and scrambled RNA (NC) were acquired from Tsingke Biotechnology Co., Ltd. China). siRNAs were transfected using Xfect RNA transfection reagent (TaKaRa, 631450) at a final concentration of 100 pM. Detailed sequences of siRNA and shRNA were listed in Table S2 in Supporting Information.

### Transcription Factors Binding to Gpx4 Promoter Region

Transcription factors binding to Gpx4 promoter region were predicted using the toolkit of Cistrome data browser (http://dbtoolkit.cistrome.org/).^[^ ^43^ ^]^ The half‐decay distance to transcription start site was set as 10 kb. Factors with RP scores > 0.80 were counted. Predicted transcription factors identified by Cistrome data browser were listed in Table S3 (Supporting Information).

### Chromatin Immunoprecipitation (ChIP)‐qPCR

ChIP‐qPCR was performed using a ChIP kit (Cell Signaling Technology, #9003S) as described before.^[^ ^35^ ^]^ Anti‐H3K9me3 (1:50, A2360, ABclonal) antibody and rabbit IgG control antibody (1:50, ABclonal, AC005) were used for ChIP‐qPCR. The following primers (5’‐3’) were used for ChIP‐qPCR: rat *Gpx4* promoter: forward, GTGGGGTTCCTGGAGAAAGG; reverse, CAGCTCCACAGGGTTGGTAG. rat *Egr1* promoter: forward, CTGACTGGTGGCCGAGTATG; reverse, ATATGGTGTTTCCGGGTCGG.

### Dual‐Luciferase Activity Assay

Dual‐luciferase activity assay was performed as described before.^[^ ^35^ ^]^ Empty control plasmids, pEGFP‐C1‐Egr1, pGL4.10‐Gpx4 promoter, and pGL4.70‐Renillawere constructed by Tsingke Biotechnology. Rat Gpx4 promoter sequence (upstream 2000 bp of transcription start site) was cloned into pGL4.10 firefly luciferase reporter vectors. Luminescence from three biological replicates was captured with a GloMax 20/20 luminometer (Promega). pGL4.70‐Renilla plasmids were used as the endogenous control.

### Ferroptosis PCR array

ADSCs were transfected with shNC or sh*Bcat1*#1. 2 d after transfection, cells were challenged with 10 µM Erastin for 6 h. The RNA was extracted from ADSCs‐shNC and ADSCs‐sh*Bcat1*#1, reverse transcribed to cDNA, mixed with qPCR SYBR, added into the Ferroptosis PCR Array 96‐well plate (WcGENE Biotech, wc‐mRNA0271‐R), and subjected to RT‐qPCR. Gene expression was calculated as 2^−∆∆Ct^ relative to *Actb* as the endogenous control.

### RNA Sequencing and Analysis

ADSCs‐shNC and ADSCs‐sh*Bcat1*#1 treated with 5 µM Erastin for 6 h were subjected to RNA sequencing (*n* = 3 biological replicates). The preparation and sequencing of RNA library were carried out by Jiayin Biotechnology Ltd. (Shanghai, China) as described before. STAR software was used for the alignment of reads to rat rn6 reference genome.^[^ ^44^ ^]^ HTSeq was used to generate read counts for individual transcripts of each sample. DESeq2 was used for the identification of Differentially expressed genes (DEGs) under a threshold of *p*‐value < 0.05 and absolute log2FC > 0.58 (FC > 1.5).^[^ ^45^ ^]^ ClusterProfiler 4.0 R package was used for Gene set enrichment analysis (GSEA).^[^ ^46^ ^]^ R version 4.1.1 was used in the present study. The raw RNA‐seq data were deposited in the Genome Sequence Archive (GSA: CRA008487).

### hADSC Injection

hADSCs (HUXMD‐01001) were purchased from Cyagen Biosciences (Guangzhou, China). After recovery of cryopreserved ADSCs, cells were cultured using the same DMEM/HG medium as rat ADSCs used. Transfection of adenoviruses carrying plasmids overexpressing EGFP into hADSCs was utilized for the evaluation of engrafted cells in livers. The numbers of cells, injection time‐point, injection sites, and evaluation procedure were the same as those in the in vivo experiments using the rat ADSCs as above described.

### Statistical Analysis

All results were presented as mean± SD. Statistical analysis was carried out using GraphPad Prism 9.0 software. For comparisons between the two groups, a two‐tailed unpaired Student's *t*‐test was performed when appropriate. For comparisons among three or more groups, one‐way or two‐way ANOVA followed by a Bonferroni post hoc test was performed. Log‐rank (Mantel‐Cox) test was used for the analysis of Kaplan‐Meier survival curves. In all cases, significance was defined as follows: *p < 0.05, **p < 0.01, *p < 0.001, and ns means not significant. The *n* values in each figure legend represent the number of the sample size or repetitions. ImageJ 1.53a was used for the quantification of histology and immunofluorescence. Detailed *p*‐values, *n*‐values, and statistical methods were listed in the corresponding figure legends.

## Conflict of Interest

The authors declare no conflict of interest.

## Supporting information

Supporting Information

Supplemental Table 4

## Data Availability

The data that support the findings of this study are available from the corresponding author upon reasonable request.

## References

[1] O. Levy , R. Kuai , E. M. J. Siren , D. Bhere , Y. Milton , N. Nissar , M. De Biasio , M. Heinelt , B. Reeve , R. Abdi , M. Alturki , M. Fallatah , A. Almalik , A. H. Alhasan , K. Shah , J. M. Karp , Sci. Adv. 2020, 6, eaba6884. 32832666 10.1126/sciadv.aba6884 PMC7439491

[2] Y. Han , J. Yang , J. Fang , Y. Zhou , E. Candi , J. Wang , D. Hua , C. Shao , Y. Shi , Signal Transduction Targeted Ther. 2022, 7, 92. 10.1038/s41392-022-00932-0 PMC8935608 35314676

[3] M. Alfaifi , Y. W. Eom , P. N. Newsome , S. K. Baik , J Hepatol 2018, 68, 1272. 29425678 10.1016/j.jhep.2018.01.030

[4] B. Lin , J. Chen , W. Qiu , K. Wang , D. Xie , X. Chen , Q. Liu , L. Peng , J. Li , Y. Mei , W. Weng , Y. Peng , H. Cao , J. Xie , S. Xie , A. P. Xiang , Z. Gao , Hepatology 2017, 66, 209. 28370357 10.1002/hep.29189

[5] X. Yang , Y. Meng , Z. Han , F. Ye , L. Wei , C. Zong , Cell Biosci. 2020, 10, 123. 33117520 10.1186/s13578-020-00480-6 PMC7590738

[6] T. Sun , F. Gao , X. Li , Y. Cai , M. Bai , F. Li , L. Du , Stem Cell Res Ther 2018, 9, 356. 30594241 10.1186/s13287-018-1098-4 PMC6311028

[7] C. Tu , R. Mezynski , J. C. Wu , Cardiovasc. Res. 2020, 116, 473. 31504255 10.1093/cvr/cvz237 PMC7252439

[8] C. M. Madl , S. C. Heilshorn , H. M. Blau , Nature 2018, 557, 335. 29769665 10.1038/s41586-018-0089-z PMC6773426

[9] P. Tsvetkov , S. Coy , B. Petrova , M. Dreishpoon , A. Verma , M. Abdusamad , J. Rossen , L. Joesch‐Cohen , R. Humeidi , R. D. Spangler , J. K. Eaton , E. Frenkel , M. Kocak , S. M. Corsello , S. Lutsenko , N. Kanarek , S. Santagata , T. R. Golub , Science 2022, 375, 1254. 35298263 10.1126/science.abf0529 PMC9273333

[10] B. R. Stockwell , J. P. Friedmann Angeli , H. Bayir , A. I. Bush , M. Conrad , S. J. Dixon , S. Fulda , S. Gascón , S. K. Hatzios , V. E. Kagan , K. Noel , X. Jiang , A. Linkermann , M. E. Murphy , M. Overholtzer , A. Oyagi , G. C. Pagnussat , J. Park , Q. Ran , C. S. Rosenfeld , K. Salnikow , D. Tang , F. M. Torti , S. V. Torti , S. Toyokuni , K. A. Woerpel , D. D. Zhang , Cell 2017, 171, 273. 28985560 10.1016/j.cell.2017.09.021 PMC5685180

[11] A. Galleu , Y. Riffo‐Vasquez , C. Trento , C. Lomas , L. Dolcetti , T. S. Cheung , M. von Bonin , L. Barbieri , K. Halai , S. Ward , L. Weng , R. Chakraverty , G. Lombardi , F. M. Watt , K. Orchard , D. I. Marks , J. Apperley , M. Bornhauser , H. Walczak , C. Bennett , F. Dazzi , Sci. Transl. Med. 2017, 9, eaam7828. 29141887 10.1126/scitranslmed.aam7828

[12] Q. Tian , C. Cao , W. Qiu , H. Wu , L. Zhou , Z. Dai , Z. Li , S. Chen , Stem Cells Int 2021, 2021, 5540149. 34840579 10.1155/2021/5540149 PMC8626202

[13] Y. Pan , J. Li , J. Wang , Q. Jiang , J. Yang , H. Dou , H. Liang , K. Li , Y. Hou , Cell Death Dis. 2022, 13, 825. 36163182 10.1038/s41419-022-05264-z PMC9512818

[14] G. E. Villalpando‐Rodriguez , S. B. Gibson , Oxid Med Cell Longev 2021, 2021, 9912436. 34426760 10.1155/2021/9912436 PMC8380163

[15] S. J. Dixon , B. R. Stockwell , Nat. Chem. Biol. 2014, 10, 9. 24346035 10.1038/nchembio.1416

[16] C. Brenner , L. Galluzzi , O. Kepp , G. Kroemer , J Hepatol 2013, 59, 583. 23567086 10.1016/j.jhep.2013.03.033

[17] M. Yan , Y. Huo , S. Yin , H. Hu , Redox Biol. 2018, 17, 274. 29753208 10.1016/j.redox.2018.04.019 PMC6006912

[18] B. R. Stockwell , Cell 2022, 185, 2401. 35803244 10.1016/j.cell.2022.06.003 PMC9273022

[19] M. Neinast , D. Murashige , Z. Arany , Annu. Rev. Physiol. 2019, 81, 139. 30485760 10.1146/annurev-physiol-020518-114455 PMC6536377

[20] W. Hou , Y. Xie , X. Song , X. Sun , M. T. Lotze , H. J. Zeh , R. Kang , D. Tang , Autophagy 2016, 12, 1425. 27245739 10.1080/15548627.2016.1187366 PMC4968231

[21] W. S. Yang , R. SriRamaratnam , M. E. Welsch , K. Shimada , R. Skouta , V. S. Viswanathan , J. H. Cheah , P. A. Clemons , A. F. Shamji , C. B. Clish , L. M. Brown , A. W. Girotti , V. W. Cornish , S. L. Schreiber , B. R. Stockwell , Cell 2014, 156, 317. 24439385 10.1016/j.cell.2013.12.010 PMC4076414

[22] M. S. Islam , T. M. Leissing , R. Chowdhury , R. J. Hopkinson , C. J. Schofield , Annu. Rev. Biochem. 2018, 87, 585. 29494239 10.1146/annurev-biochem-061516-044724

[23] W. Xu , H. Yang , Y. Liu , Y. Yang , P. Wang , S. H. Kim , S. Ito , C. Yang , P. Wang , M. T. Xiao , L. X. Liu , W. Q. Jiang , J. Liu , J. Y. Zhang , B. Wang , S. Frye , Y. Zhang , Y. H. Xu , Q. Y. Lei , K. L. Guan , S. M. Zhao , Y. Xiong , Cancer Cell 2011, 19, 17. 21251613 10.1016/j.ccr.2010.12.014 PMC3229304

[24] R. Chowdhury , K. K. Yeoh , Y.‐M. Tian , L. Hillringhaus , E. A. Bagg , N. R. Rose , I. K. H. Leung , X. S. Li , E. C. Y. Woon , M. Yang , M. A. McDonough , O. N. King , I. J. Clifton , R. J. Klose , T. D. W. Claridge , P. J. Ratcliffe , C. J. Schofield , A. Kawamura , EMBO Rep. 2011, 12, 463. 21460794 10.1038/embor.2011.43 PMC3090014

[25] V. Volarevic , J. Nurkovic , N. Arsenijevic , M. Stojkovic , Stem Cells 2014, 32, 2818. 25154380 10.1002/stem.1818

[26] X. He , W. Hong , J. Yang , H. Lei , T. Lu , C. He , Z. Bi , X. Pan , Y. Liu , L. Dai , W. Wang , C. Huang , H. Deng , X. Wei , Signal Transduction Targeted Ther. 2021, 6, 270. 10.1038/s41392-021-00688-z PMC8280232 34262012

[27] S. J. Dixon , K. M. Lemberg , M. R. Lamprecht , R. Skouta , E. M. Zaitsev , C. E. Gleason , D. N. Patel , A. J. Bauer , A. M. Cantley , W. S. Yang , B. Morrison 3rd , B. R. Stockwell , Cell 2012, 149, 1060. 22632970 10.1016/j.cell.2012.03.042 PMC3367386

[28] J. Chen , X. Li , C. Ge , J. Min , F. Wang , Cell Death Differ. 2022, 29, 467. 35075250 10.1038/s41418-022-00941-0 PMC8901678

[29] A. Ichihara , E. Koyama , J. Biochem. 1966, 59, 160. 5943594 10.1093/oxfordjournals.jbchem.a128277

[30] S. Raffel , M. Falcone , N. Kneisel , J. Hansson , W. Wang , C. Lutz , L. Bullinger , G. Poschet , Y. Nonnenmacher , A. Barnert , C. Bahr , P. Zeisberger , A. Przybylla , M. Sohn , M. Tönjes , A. Erez , L. Adler , P. Jensen , C. Scholl , S. Fröhling , S. Cocciardi , P. Wuchter , C. Thiede , A. Flörcken , J. Westermann , G. Ehninger , P. Lichter , K. Hiller , R. Hell , C. Herrmann , et al., Nature 2017, 551, 384. 29144447 10.1038/nature24294

[31] Z. Zhu , A. Achreja , N. Meurs , O. Animasahun , S. Owen , A. Mittal , P. Parikh , T. W. Lo , J. Franco‐Barraza , J. Shi , V. Gunchick , M. H. Sherman , E. Cukierman , A. M. Pickering , A. Maitra , V. Sahai , M. A. Morgan , S. Nagrath , T. S. Lawrence , D. Nagrath , Nat Metab 2020, 2, 775. 32694827 10.1038/s42255-020-0226-5 PMC7438275

[32] W. Zhang , J. Li , K. Suzuki , J. Qu , P. Wang , J. Zhou , X. Liu , R. Ren , X. Xu , A. Ocampo , T. Yuan , J. Yang , Y. Li , L. Shi , D. Guan , H. Pan , S. Duan , Z. Ding , M. Li , F. Yi , R. Bai , Y. Wang , C. Chen , F. Yang , X. Li , Z. Wang , E. Aizawa , A. Goebl , R. D. Soligalla , P. Reddy , et al., Science 2015, 348, 1160. 25931448 10.1126/science.aaa1356 PMC4494668

[33] S. Bhattacharyya , M. Wu , F. Fang , W. Tourtellotte , C. Feghali‐Bostwick , J. Varga , Matrix Biol. 2011, 30, 235. 21511034 10.1016/j.matbio.2011.03.005 PMC3135176

[34] E. El Agha , R. Kramann , R. K. Schneider , X. Li , W. Seeger , B. D. Humphreys , S. Bellusci , Cell Stem Cell 2017, 21, 166. 28777943 10.1016/j.stem.2017.07.011

[35] F. Zhang , G. Hu , X. Chen , L. Zhang , L. Guo , C. Li , H. Zhao , Z. Cui , X. Guo , F. Sun , D. Song , W. Yan , Y. Xia , S. Wang , M. Fan , L. Tao , Signal Transduction Targeted Ther. 2022, 7, 1. 10.1038/s41392-022-00971-7 PMC9163108 35654769

[36] D.‐N. Su , S.‐P. Wu , S.‐Z. Xu , Stem Cell Res Ther 2020, 11, 395. 32928296 10.1186/s13287-020-01911-4 PMC7489041

[37] W. Yan , Y. Guo , L. Tao , W. B. Lau , L. Gan , Z. Yan , R. Guo , E. Gao , G. W. Wong , W. L. Koch , Y. Wang , X. L. Ma , Circulation 2017, 136, 2162. 28978553 10.1161/CIRCULATIONAHA.117.029557 PMC5705403

[38] F. Zhang , S. Zhao , W. Yan , Y. Xia , X. Chen , W. Wang , J. Zhang , C. Gao , C. Peng , F. Yan , H. Zhao , K. Lian , Y. Lee , L. Zhang , W. B. Lau , X. Ma , L. Tao , EBioMedicine 2016, 13, 157. 27843095 10.1016/j.ebiom.2016.10.013 PMC5264279

[39] Y. Li , Z. Xiong , W. Yan , E. Gao , H. Cheng , G. Wu , Y. Liu , L. Zhang , C. Li , S. Wang , M. Fan , H. Zhao , F. Zhang , L. Tao , Theranostics 2020, 10, 5623. 32373236 10.7150/thno.44836 PMC7196282

[40] T. Hirayama , S. Kadota , M. Niwa , H. Nagasawa , Metallomics 2018, 10, 794. 29863204 10.1039/c8mt00049b

[41] J. Cox , M. Mann , Nat. Biotechnol. 2008, 26, 1367. 19029910 10.1038/nbt.1511

[42] K. Lian , C. Du , Y. Liu , D. Zhu , W. Yan , H. Zhang , Z. Hong , P. Liu , L. Zhang , H. Pei , J. Zhang , C. Gao , C. Xin , H. Cheng , L. Xiong , L. Tao , Diabetes 2015, 64, 49. 25071024 10.2337/db14-0312

[43] R. Zheng , C. Wan , S. Mei , Q. Qin , Q. Wu , H. Sun , C. H. Chen , M. Brown , X. Zhang , C. A. Meyer , X. S. Liu , Nucleic Acids Res. 2019, 47, D729. 30462313 10.1093/nar/gky1094 PMC6324081

[44] A. Dobin , C. A. Davis , F. Schlesinger , J. Drenkow , C. Zaleski , S. Jha , P. Batut , M. Chaisson , T. R. Gingeras , Bioinformatics 2013, 29, 15. 23104886 10.1093/bioinformatics/bts635 PMC3530905

[45] M. I. Love , W. Huber , S. Anders , Genome Biol. 2014, 15, 550. 25516281 10.1186/s13059-014-0550-8 PMC4302049

[46] T. Wu , E. Hu , S. Xu , M. Chen , P. Guo , Z. Dai , T. Feng , L. Zhou , W. Tang , L. Zhan , X. Fu , S. Liu , X. Bo , G. Yu , The Innovation 2021, 2. 10.1016/j.xinn.2021.100141 PMC8454663 34557778

