# Supplementary material for: Suppressing Mesenchymal Stromal Cell Ferroptosis Via Targeting a Metabolism‐Epigenetics Axis Corrects their Poor Retention and Insufficient Healing Benefits in the Injured Liver Milieu
Source: Adv Sci (Weinh). 2023 Feb 19;10(13):2206439. doi: 10.1002/advs.202206439 (PMC10161111; doi:10.1002/advs.202206439)
Supplement: Supplementary file 1 — Supporting Information [file ADVS-10-2206439-s002.pdf]

ADVANCED SCIENCE
Open Access

Supporting Information

for *Adv. Sci.*, DOI 10.1002/advs.202206439

Suppressing Mesenchymal Stromal Cell Ferroptosis Via Targeting a Metabolism-Epigenetics Axis Corrects their Poor Retention and Insufficient Healing Benefits in the Injured Liver Milieu

Guangyu Hu, Zhe Cui, Xiyao Chen, Fangfang Sun, Tongzheng Li, Congye Li, Ling Zhang, Xiong Guo, Hang Zhao, Yunlong Xia, Wenjun Yan, Wei Yi, Miaomiao Fan, Rongjin Yang, Shan Wang, Ling Tao and Fuyang Zhang**

Supporting Information

Suppressing mesenchymal stromal cell ferroptosis via targeting a metabolism-epigenetics axis corrects their poor retention and insufficient healing benefits in the injured liver milieu

Guangyu Hu, Zhe Cui, Xiyao Chen, Fangfang Sun, Tongzheng Li, Congye Li, Ling Zhang, Xiong Guo, Hang Zhao, Yunlong Xia, Wenjun Yan, Miaomiao Fan, Rongjin Yang, Shan Wang, Ling Tao, Fuyang Zhang**

E-mail: lingtao@fmmu.edu.cn (to Ling Tao); plazhangfuyang@163.com (to Fuyang Zhang)

This Word file includes:

Figure S1 to S12
Table S1 to S4

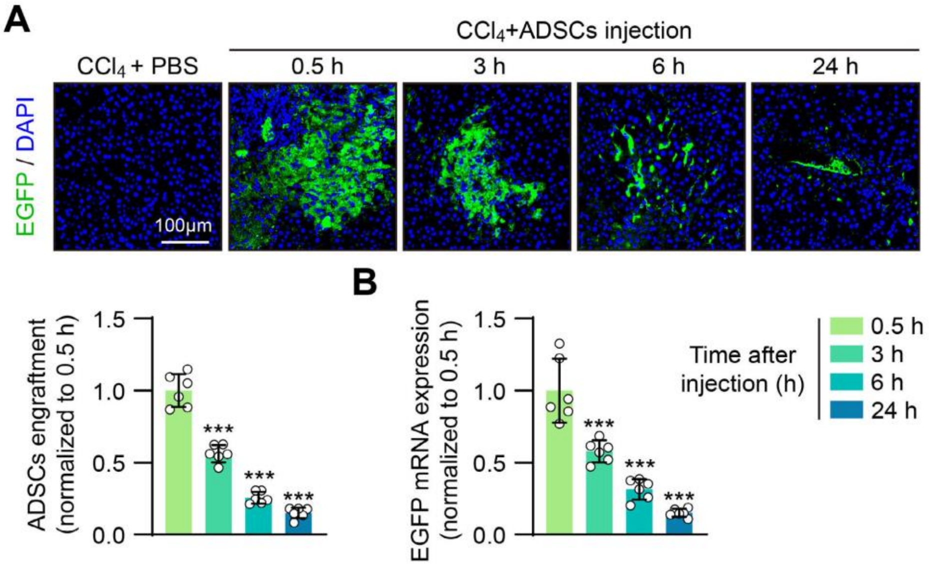

Figure S1. MSCs undergo rapid depletion after implantation into CCl_4-injured livers. (**A** and **B**) Engraftment time course at a period of 24 h of EGFP-labeled ADSCs in the CCl_4 injured livers as determined by immunostaining of EGFP (A) and RT-qPCR of EGFP mRNA levels (B). The CCl_4 + PBS group was used as negative control. The cell engraftment at 0.5 h was normalized as 1. (n = 6 rats per group). Data are presented as mean ± SD. Data were analyzed by one-way ANOVA followed by a Bonferroni post hoc test. *P < 0.05, **P < 0.01, and ***P < 0.001.

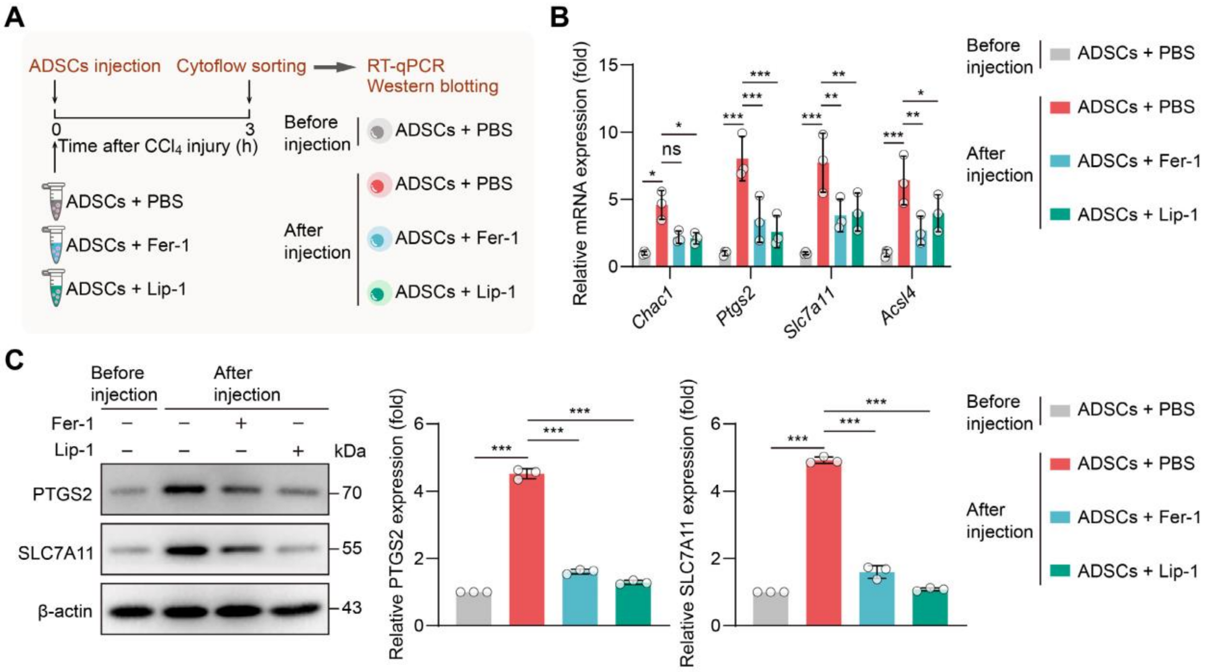

Figure S2. MSCs express ferroptosis-associated markers after implantation into CCl_4-injured livers. (**A**) Illustration of animal models. The CCl4-injured livers were injected with EGFP-labeled ADSCs supplemented with PBS, Fer-1, or Lip-1 in the solvent. Fer-1 and Lip-1 were dissolved in 200 µl PBS solvent at a final concentration of 2 µM. Extra ADSCs supplemented with PBS were preserved as the control for the following experiments. 3 h after injection, EGFP-labeled ADSCs were sorted out from the injured livers using flow cytometry. ADSCs before injection and after injection were subjected to RT-qPCR and Western blot analysis. (**B**) the mRNA expression of ferroptosis-associated markers *Chac1*, *Ptgs2*, *Slc7a11*, and *Acsl4* in ADSCs before and after injection as determined by RT-qPCR. ADSCs + PBS before injection group was normalized as 1 (n = 3 rats per group). (**C**) Western blot analysis of PTGS2 and SLC7A11 protein expression in ADSCs before and after injection (n = 3 technical replicates). Data are presented as mean ± SD. Data were analyzed by one-way ANOVA followed by a Bonferroni post hoc test. *$P < 0.05$, **$P < 0.01$, ***$P < 0.001$ and ns means not significant.

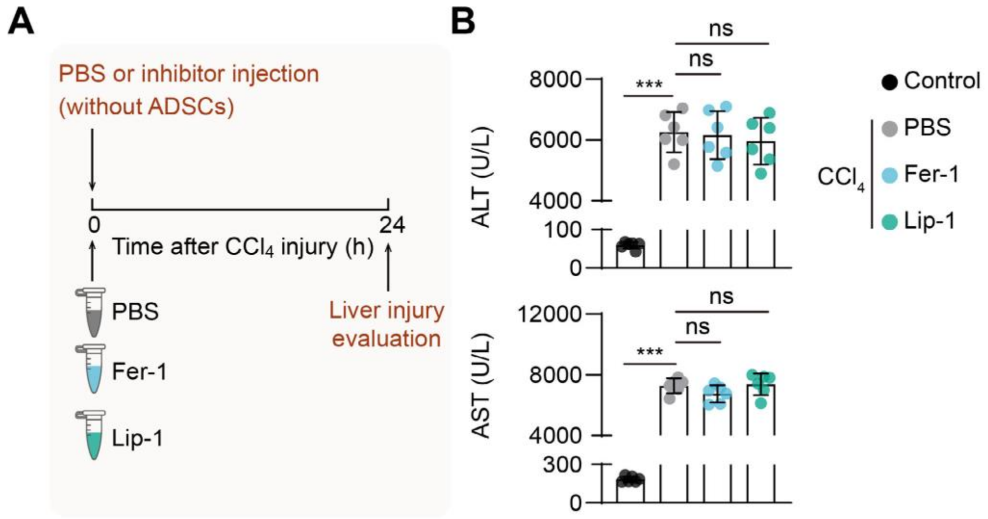

Figure S3. Fer-1 or Lip1 alone in the solvent does not alleviate CCl_4-induced liver injury. (**A**) Illustration of animal models. The CCl_4 injured livers were treated with PBS, Fer-1, or Lip-1 alone without ADSCs. Fer-1 and Lip-1 were dissolved in 200 µl PBS solvent at a final concentration of 2 µM. (**B**) Serum ALT and AST levels at 24 h post liver injury (n = 6 rats per group). Data are presented as mean ± SD. Data were analyzed by one-way ANOVA followed by a Bonferroni post hoc test. *P < 0.05, **P < 0.01, ***P < 0.001, and ns means not significant.

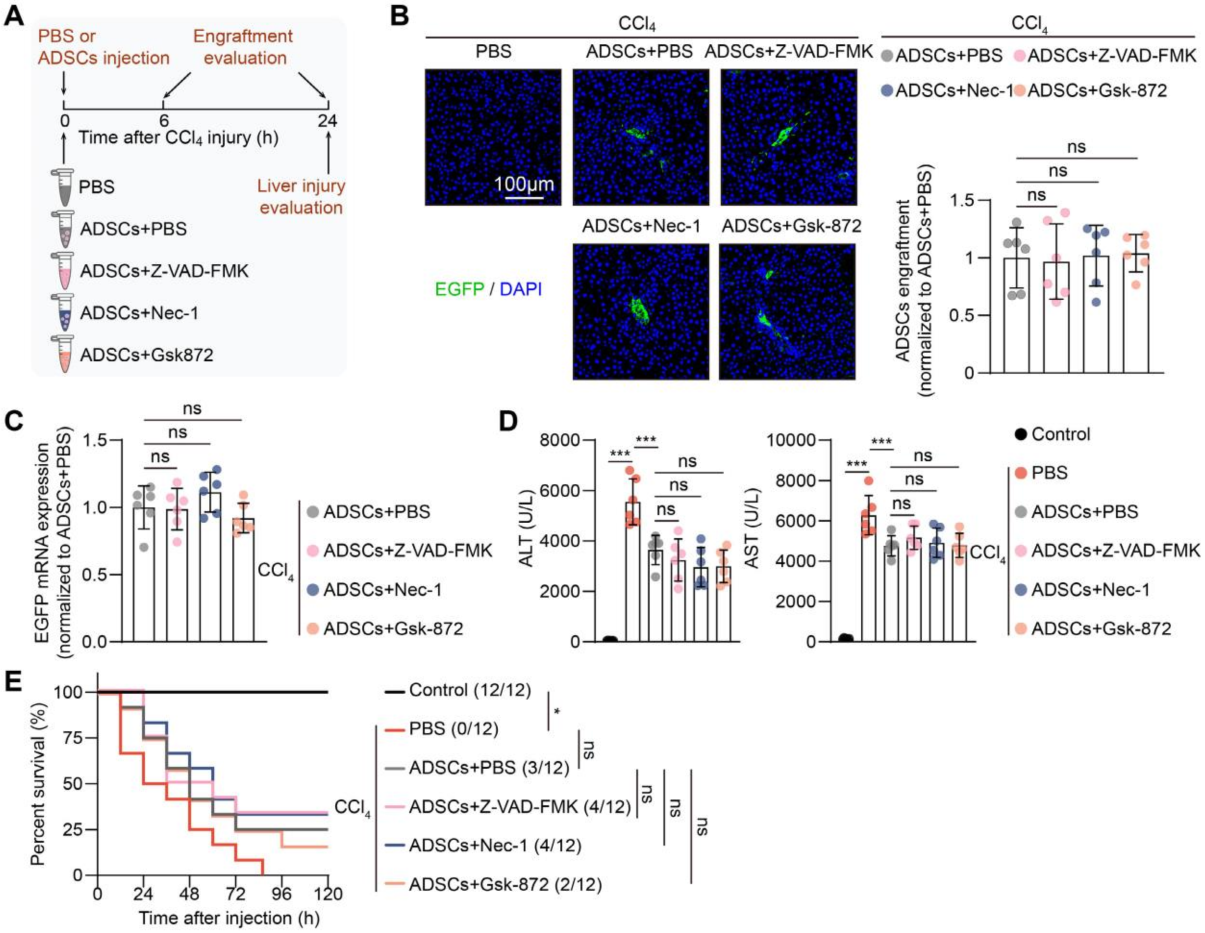

Figure S4. Z-VAD-FMK, Nec-1, or GSK-872 supplemented in the cell solvent does not enhance MSC retention and therapeutic efficacy in the injured livers. (**A**) Illustration of animal models. The CCl_4 injured livers were treated with PBS, ADSCs + PBS, ADSCs + Z-VAD-FMK, ADSCs + Nec-1, or ADSCs + Gsk-872. Z-VAD-FMK, Nec-1, or GSK-872 was dissolved in the PBS solvent at a final concentration of 50 µM, 10 µM, or 10 µM, respectively. (**B** and **C**) Engraftment evaluation of EGFP-labeled ADSCs at 24 h post injection. Engraftment was evaluated by immunostaining of EGFP (B)and RT-qPCR of EGFP mRNA levels (C). The CCl_4 + PBS group was used as negative control. The cell engraftment of ADSCs + PBS was normalized as 1 (n = 6 rats per group). (**D**) Serum ALT and AST levels at 24 h post CCl_4 injury (n = 6 rats per group). (**E**) Kaplan-Meier survival curves of CCl_4-injured rats. (n = 12 rats per group). Data are presented as mean ± SD. Data shown in (B), (C), and (D) were analyzed by one-way ANOVA followed by a Bonferroni post hoc test. Data shown in (E) were analyzed by log-rank Mantel-Cox test. *P < 0.05, **P < 0.01, ***P < 0.001, and ns means not significant.

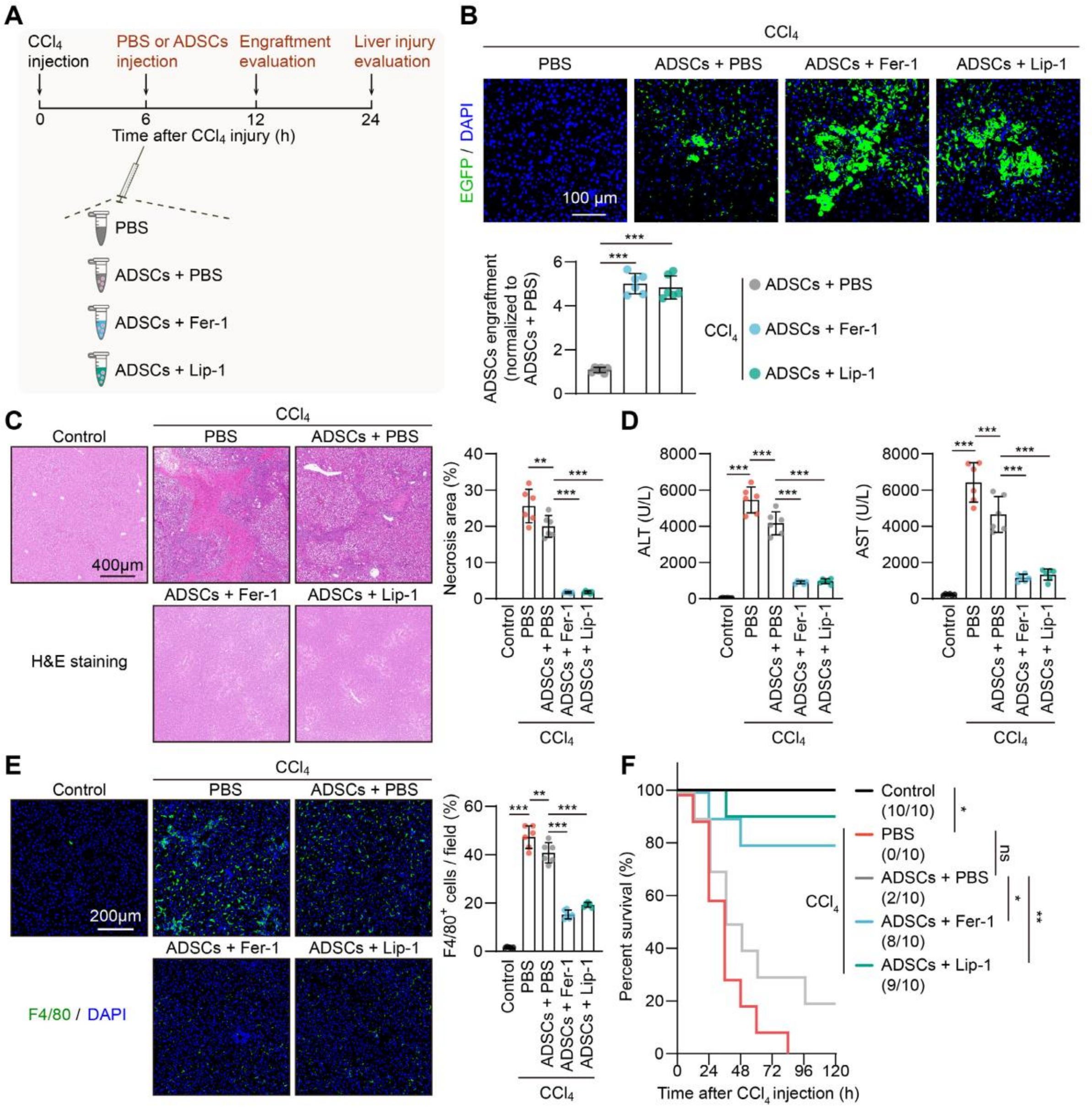

Figure S5. Both Fer-1 and Lip1 in the cell solvent increase the retention and liver-protection effects of MSCs injected at 6 h after CCl_4 injury. (**A**) Illustration of animal models. Rats were injected with PBS (Control) or CCl_4. 6 h after CCl_4 injection, rat livers were administered with PBS, ADSCs + PBS, ADSCs + Fer-1, or ADSCs + Lip-1. Fer-1 and Lip-1 were dissolved in the PBS solvent at a final concentration of 2 μM. (**B**) Top panel, representative images of EGFP-labeled ADSCs (green) at 12 h post CCl_4 injection as determined by immunostaining of EGFP. The CCl_4 + PBS group was used as negative control. Bottom panel, quantification of EGFP-labeled ADSCs in the liver sections (*n* = 6 rats per group). (**C**) Left panel, representative images of H&E staining of liver sections at 24 h post CCl_4 injury. Right panel, quantification of necrosis area (%) based on H&E staining (*n* = 6 rats per group). (**D**) Serum ALT and AST levels at 24 h post CCl_4 injury (*n* = 6 rats per group). (**E**) Left panel, representative images of immunostaining of F4/80 (green) in liver sections at 24 h post CCl_4 injury. Right panel, quantification of $F4/80^+$ cells per field. (*n* = 6 rats per group). Percent means the proportions of F4/80-positive cells to the total DAPI-positive cells per field. (**F**) Kaplan-Meier survival curves of CCl_4-injured rats (*n* = 10 rats per group). Data are presented as mean ± SD. Data shown in (B), (C), (D), and (E) were analyzed by one-way ANOVA followed by a Bonferroni post hoc test. Data shown in (F) were

analyzed by log-rank Mantel-Cox test. *P < 0.05, **P < 0.01, ***P < 0.001, and ns means not significant.

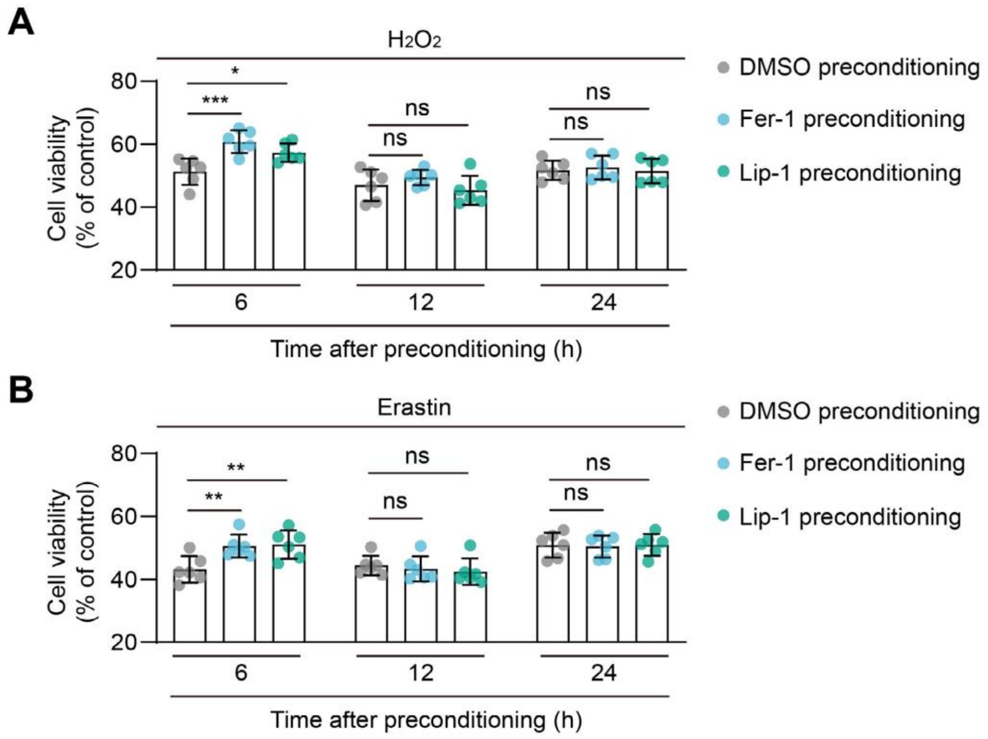

Figure S6. Fer-1 or Lip1 preconditioning minimally alleviates *in vitro* cell death of MSCs upon ROS or Erastin stress. (**A** and **B**) ADSCs were preconditioned with DMSO, Fer-1 (2 μM), or Lip-1 (2 μM) for 24 h and then changed to the fresh medium. At 6, 12, or 24 h after preconditioning, the cells were treated with H_2O_2 (200 μM, A) or Erastin (10 μM, B) for 6 h followed by cell viability analysis (n = 6 biological replicates). The experimental group that was given the same intervention but with the vehicle (PBS or DMSO) instead of H_2O_2 or Erastin was set as the control group, and the cell viability of the control group was set at 100%. Data are presented as mean ± SD. Data were analyzed by one-way ANOVA followed by a Bonferroni post hoc test. *P < 0.05, **P < 0.01, ***P < 0.001, and ns means not significant.

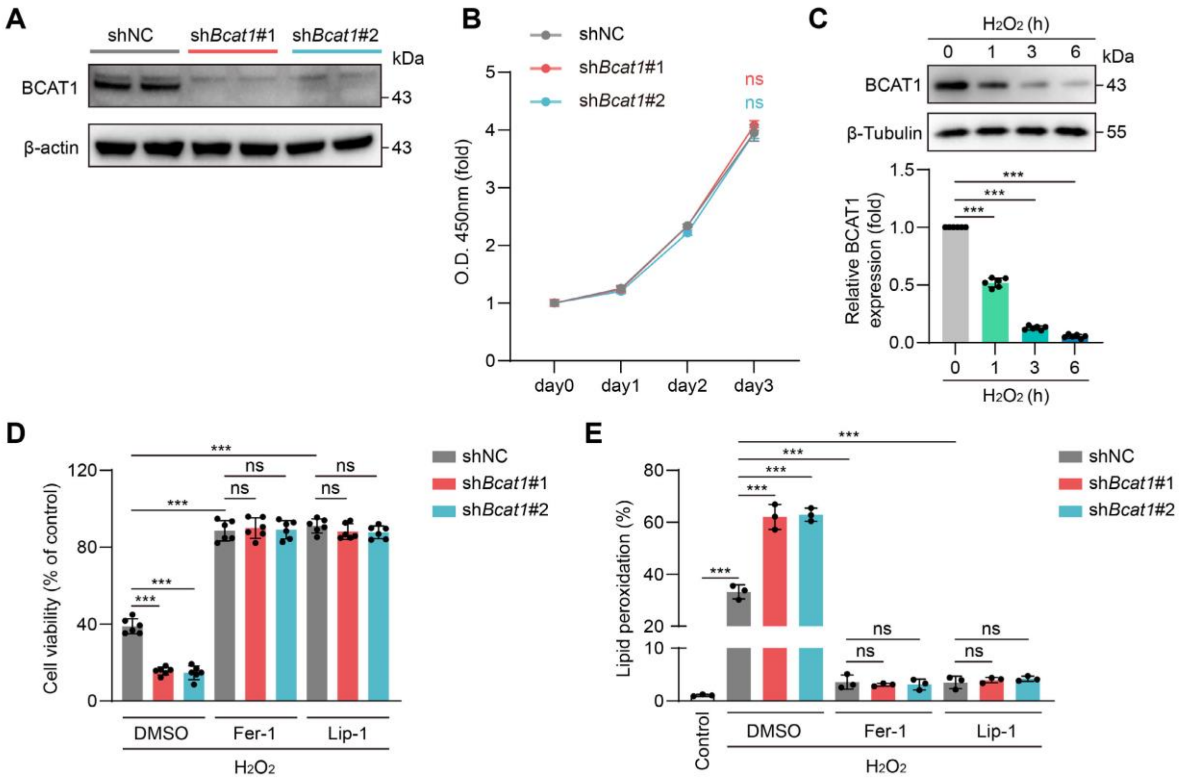

Figure S7. BCAT1 downregulation sensitizes MSCs to ferroptotic cell death upon ROS stress. (**A**) Western blot analysis of BCAT1 protein expression in ADSCs transfected with shNC, sh*Bcat1*#1, or sh*Bcat1*#2. (**B**) Cell proliferation assay of ADSCs transfected with shNC, sh*Bcat1*#1, or sh*Bcat1*#2 as determined by CCK-8. CCK-8 was added in the medium at 2 d after transfection and detected for 4 consecutive d. The first day CCK-8 was added in the medium was set as 'day 0' and normalized as 1 in each group(n = 6 biological replicates). (**C**) ADSCs were treated with H_2O_2 (200 μM) for 0, 1, 3, or 6 h. BCAT1 protein expression was detected at each time point by Western blot analysis (n = 6 biological replicates). (**D**) ADSCs were transfected with shNC, sh*B*cat1#1, or sh*Bcat1*#2 upon H_2O_2 (200 μM) treatment in the presence of DMSO, Fer-1 (2 μM), or Lip-1 (2 μM) for 6 h followed by cell viability analysis (n = 6 biological replicates). The experimental group that was given the same intervention but with the vehicle (PBS) instead of H_2O_2 was set as the control group, and the cell viability of the control group was set at 100%. (**E**) ADSCs were transfected with shNC, sh*B*cat1#1, or sh*Bcat1*#2 upon H_2O_2 (200 μM) treatment in the presence of DMSO, Fer-1 (2 μM), or Lip-1（2 μM）for 6 h followed by lipid peroxidation analysis using BODIPY 581/591 C11 staining (n = 3 biological replicates). Data are presented as mean ± SD. Data in (B) and (C) were analyzed by one-way ANOVA followed by a Bonferroni post hoc test. Data in (D) and (E) were analyzed by two-way ANOVA followed by a Bonferroni post hoc test. *P < 0.05, **P < 0.01, ***P < 0.001, and ns means not significant.

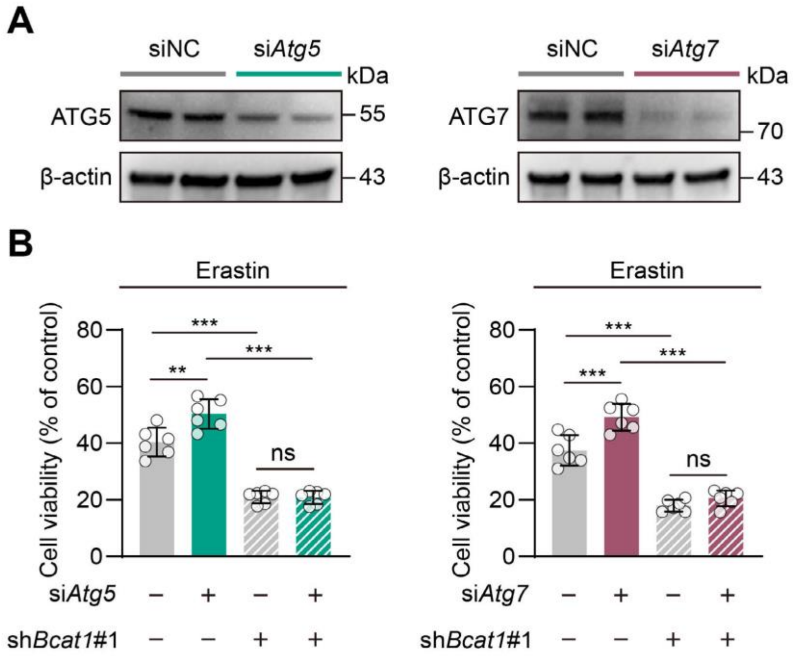

Figure S8. Suppression of ATG5 or ATG7 does not rescue the exacerbated ferroptotic cell death due to BCAT1 downregulation in MSCs. (**A**) Western blot analysis of ATG5 (left) and ATG7 (right) protein expression in ADSCs transfected with siNC, si*Atg5*, or si*Atg7*, respectively. (**B**) Cell viability analysis in ADSCs transfected with shNC or sh*Bcat1*#1,co-transfected with siNC, si*Atg5*, or si*Atg7* upon DMSO (Control) or Erastin (10 μM, 6 h) treatment (n = 6 biological replicates). Data are presented as mean ± SD. The experimental group that was given the same intervention but with the vehicle (DMSO) instead of Erastin was set as the control group, and the cell viability of the control group was set at 100%. Data were analyzed by two-way ANOVA followed by a Bonferroni post hoc test. *P < 0.05, ** P < 0.01, and ***P < 0.001.

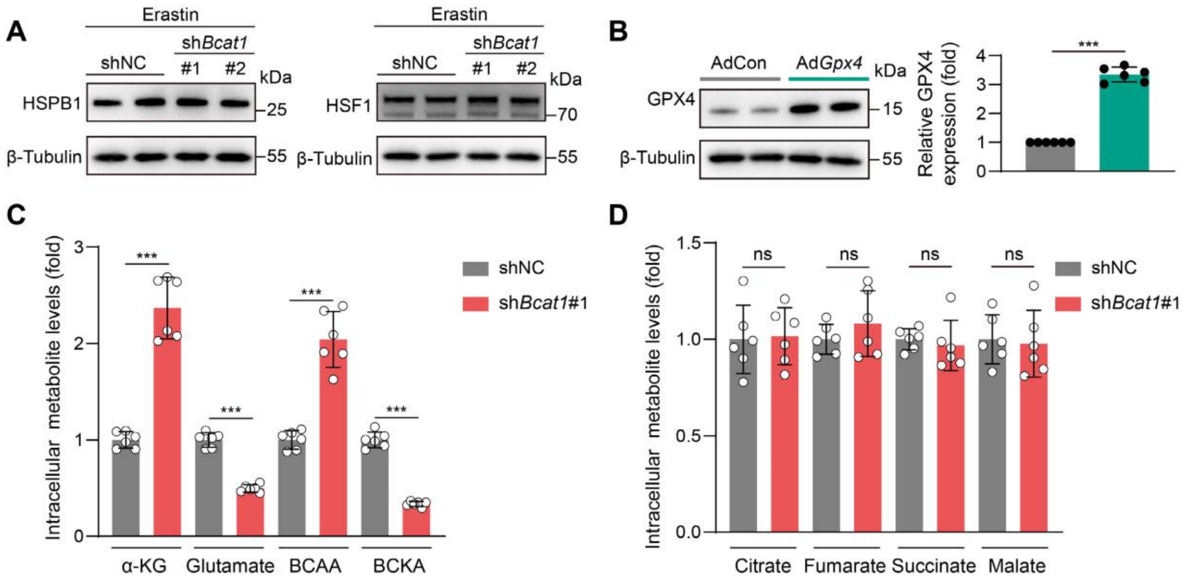

Figure S9. BCAT1 downregulation causes intracellular α-KG accumulation. (**A**) Western blot analysis of HSPB1 and HSF1 protein expression in ADSCs transfected with shNC or sh*Bcat1*#1 upon Erastin (10 μM) treatment for 6 h. (**B**) ADSCs were transfected with control vectors (AdCon) or GPX4 overexpression vectors (Ad*Gpx4*), followed by Western blot analysis(n = 6 biological replicates). (**C**) Measurement of intracellular concentration of α-KG, Glutamate, BCAA, and BCKA in ADSCs transfected with shNC or sh*Bcat1*#1. Metabolite levels in shNC group was normalized as 1 (n = 6 biological replicates). (**D**) Measurement of intracellular concentration of citrate, fumarate, succinate, and malate in ADSCs transfected with shNC or sh*Bcat1*#1. Metabolite levels in shNC group was normalized as 1 (n = 6 biological replicates). Data are presented as mean ± SD. Data were analyzed by two-tailed unpaired Student's *t*-test. $^{*}P < 0.05$, $^{**}P < 0.01$, $^{***}P < 0.001$, and ns means not significant.

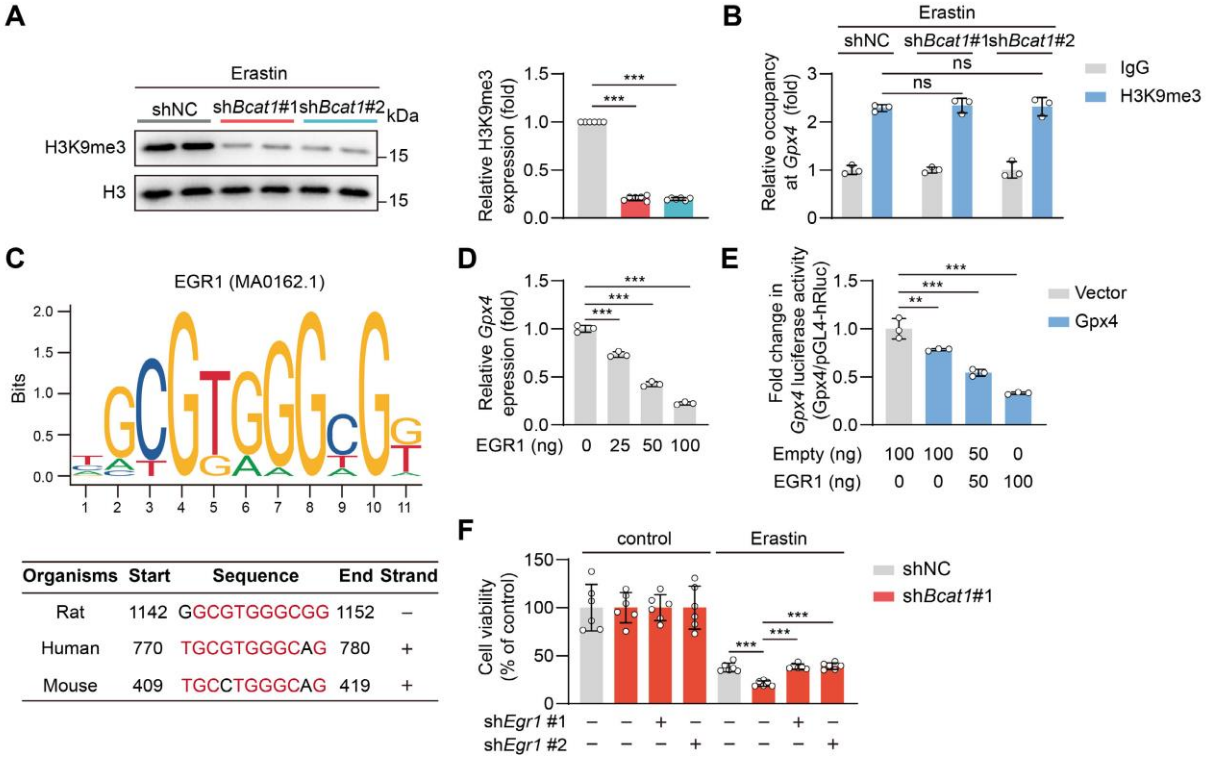

Figure S10. EGR1 upregulation mediated by H3K9me3 downregulation directly suppresses GPX4 transcription. (**A**) Western blot analysis (left) and corresponding quantification (right) of H3K9me3 protein expression in ADSCs transfected with shNC, sh*Bcat1*#1, or sh*Bcat1*#2 upon Erastin (10 μM) treatment for 6 h (*n* = 6 biological replicates). (**B**) H3K9me3 occupancy at *Gpx4* promoter region in ADSCs transfected with shNC, sh*Bcat1*#1 or sh*Bcat1*#2 upon Erastin (10 μM, 6 h) treatment as determined by ChIP-qPCR (*n* = 3 biological replicates). (**C**) EGR1 binding motif (top) and predicted binding region at *Gpx4* promoter (bottom) in rat, human, and mouse. . (**D**) ADSCs were transfected with 0, 25, 50, or 100 ng vectors overexpressing EGR1. 2 d post transfection, mRNA levels of *Gpx4* were analyzed by RT-qPCR. *Actb* was used as the endogenous control gene (*n* = 3 biological replicates). (**E**) ADSCs were transfected with firefly luciferase reporter driven by the *Gpx4* promoter sequence and co-transfected with indicated empty vectors or vectors overexpressing EGR1. Renilla luciferase activity was used as a transfection control (*n* = 3 biological replicates). (**F**) Cell viability analysis in ADSCs transfected with shNC or sh*Bcat1*#1, co-transfected with shNC, sh*Egr1*#1, or sh*Egr1*#2 upon DMSO (Control) or Erastin (10 μM, 6 h) treatment (*n* = 6 biological replicates). Data are presented as mean ± SD. Data shown in (A), (D), (E), and (F) were analyzed by one-way ANOVA followed by a Bonferroni post hoc test. Data shown in (B) were analyzed by two-way ANOVA followed by a Bonferroni post hoc test. *$P < 0.05$, ** $P < 0.01$, ***$P < 0.001$, and ns means not significant.

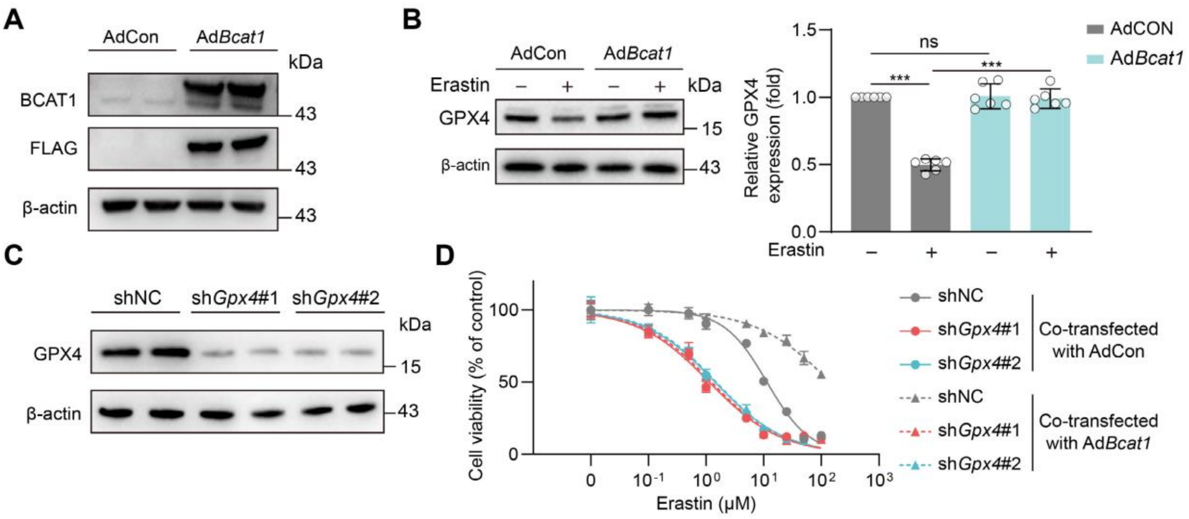

Figure S11. Overexpression of BCAT1 prevents MSCs from ferroptosis in a GPX4 dependent manner. (**A**) ADSCs were transfected with empty adenovirus vectors (AdCon) or BCAT1 overexpression adenovirus vectors (Ad*Bcat1*), followed by Western blot analysis 2 d post transfection. Overexpressed BCAT1 was merged with a 3× Flag tag. (**B**) Western blot analysis (left) and corresponding quantification (right) of GPX4 protein expression in ADSCs transfected with AdCon or Ad*Bcat1* with or without Erastin (10 μM) treatment for 6 h (n = 6 biological replicates). (**C**) ADSCs were transfected with scrambled shRNA(shNC) or shRNA targeting *Gpx4* (sh*Gpx4*#1 and sh*Gpx4*#2), followed by Western blot analysis 2 d post-transfection. (**D**) Cell viability analysis of ADSCs upon increasing concentrations of Erastin treatment. ADSCs were transfected with shNC, sh*Gpx4*#1 or sh*Gpx4*#2 and co-transfected with either AdCon or Ad*Bcat1*. Data are presented as mean ± SD. Data shown in (B) were analyzed by two-way ANOVA followed by a Bonferroni post hoc test. *P < 0.05, ** P < 0.01, ***P < 0.001, and ns means not significant.

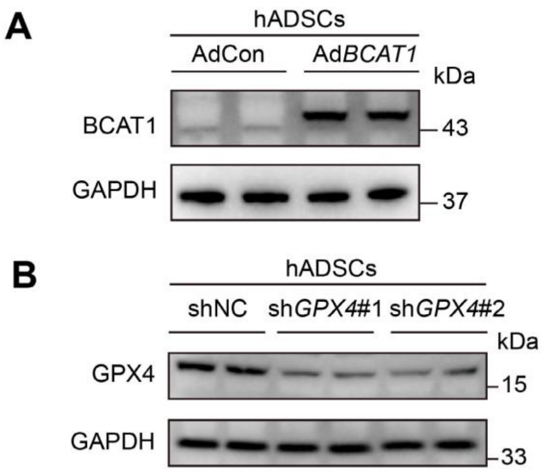

Figure S12. BCAT1 Overexpression and GPX4 knockdown in hADSCs. (**A**) Human ADSCs (hADSCs) were transfected with empty adenovirus vectors (AdCon) or human BCAT1 overexpression adenovirus vectors (Ad*BCAT1*), followed by Western blot analysis 2 d post-transfection. Overexpressed BCAT1 was merged with a 3× Flag tag. (**B**) hADSCs were transfected with scrambled shRNA (shNC) or shRNA targeting human *GPX4* (sh*GPX4*#1 and sh*GPX4*#2), followed by Western blot analysis 2 d post-transfection.

Table S1. Primers for RT-qPCR (5'-3').

Genes	Forward	Reverse	Organism
Il6	GCAAGAGACTTCCAGCCAGT	TCTGACAGTGCATCATCGCT	Rat
Il1b	CAGAACATAAGCCAACAAGTGGT	GATTCTTCCCCTTGAGGCCC	Rat
Ifng	AGTGTCATCGAATCGCACCT	TGTGGGTTGTTCACCTCGAA	Rat
Tnf	GATCGGTCCCAACAAGGAGG	CTTGGTGGTTTGCTACGACG	Rat
Chac1	TGGATTTTCGGGTACGGCTC	TGGATTTTCGGGTACGGCTC	Rat
Ptgs2	TACGAAGACCCTGCCTACGA	GGGTGGGCTTCAGCAGTAAT	Rat
Slc7a11	TCGTCCTTTCAAGGTGCCTC	GGCAGATGGCCAAGGATTTG	Rat
Acsl4	GATTCTTCCCCTTGAGGCCC	GATTCTTCCCCTTGAGGCCC	Rat
Actb	ATCATTGCTCCTCCTGAGCG	CGCAGCTCAGTAACAGTCCG	Rat

Table S2. siRNA and shRNA sequences (5'-3').

Name	Forward or Top strand	Reverse or Bottom strand	Organism
si*Uba3*	AGAUAUCAGCAUUCCAUUGAU	CAAUGGAAUGCUGAUAUCUCU	Rat
si*Nmd3*	AACUUUACUUCGUAAACAGGC	CUGUUUACGAAGUAAAGUUGA	Rat
si*Uba2*	AUGUUUCUUUUGAAACAAGAA	CUUGUUUCAAAAGAAACAUGU	Rat
si*Bcat1*	CGCAUCAUGGCAAAGUUGACUGAUA	UAUCAGUCAACUUUGCCAUGAUGCG	Rat
si*Lta4h*	AUAUUUCGUGUGCAAUAACAU	GUUAUUGCACACGAAAUAUCG	Rat
si*Eno2*	AAAUCCAAGUCGUAUUUGCCA	GCAAAUACGACUUGGAUUUCA	Rat
si*Pak1*	UUUAUCUCCAGCUAAGAUGGA	CAUCUUAGCUGGAGAUAAAAC	Rat
si*Eif2a*	ACUUCAUUGUUAACAUUCCGG	GGAAUGUUAACAAUGAAGUUC	Rat
si*Ddt*	AGUUUGUUUCCAACUCAACGA	GUUGAGUUGGAAACAAACUUG	Rat
si*Fabp3*	UCUUUAUGGUGAUGGUAUCCC	GAUACCAUCACCAUAAAGACA	Rat
si*Atg5*	UCUGAAAGUGCUUUUUCACUU	GUGAAAAAGCACUUUCAGAAG	Rat
si*Atg7*	UACUUGAAUAAGAAGUAUGGC	CAUACUUCUUAUUCAAGUACG	Rat
siNC	UUCUCCGAACGUGUCACGUTT	ACGUGACACGUUCGGAGAATT	Rat
sh*Bcat1*#1	TCGAGGCGCATCATGGCAAAGTTGACTGATATTCAAGAGATATCAGTCAACTTTGCCATGATGCGTTTTTTA	AGCTTAAAAAACGCATCATGGCAAAGTTGACTGATATCTCTTGAATATCAGTCAACTTTGCCATGATGCGCC	Rat
sh*Bcat1*#2	TCGAGGCACATCACCATGGATGACCTGTCCATTCAAGAGATGGACAGGTCATCCATGGTGATGTGTTTTTTA	AGCTTAAAAAACACATCACCATGGATGACCTGTCCATCTCTTGAATGGACAGGTCATCCATGGTGATGTGCC	Rat
sh*Gpx4*	TCGAGTATCCAGGCAAACCATGUGCCTTCAAGAGACACATGGTTTGCCUGGATAAGTTTTTTA	AGCTTAAAAAATATCCAGGCAAACCATGUGCCTCTCTTGAACACATGGTTTGCCUGGATAAGC	Rat
sh*Egr*#1	TCGAGGCTGGTGGAGACAAGTTATTTCAAGAGAATAACTTGTCTCCACCAGCTTTTTTA	AGCTTAAAAAAGCTGGTGGAGACAAGTTATTCTCTTGAAATAACTTGTCTCCACCAGCC	Rat
sh*Egr*#2	TCGAGGCCTTGTGAGCATGACCAATTCAAGAGATTGGTCATGCTCACAAGGCTTTTTTA	AGCTTAAAAAAGCCTTGTGAGCATGACCAATCTCTTGAATTGGTCATGCTCACAAGGCC	Rat

sh*GPX4* #1	TCGAGGTGAGGCAAGACCGAAGTAAATTCAAGAGATTTACTTCGGTCTTGCCTCACTTTTTTA	AGCTTAAAAAAGTGAGGCAAGACCGAAGTAAATCTCTTGAATTTACTTCGGTCTTGCCTCACC	Human
sh*GPX4* #2	TCGAGGTGGATGAAGATCCAACCCAATTCAAGAGATTGGGTTGGATCTTCATCCACTTTTTTA	AGCTTAAAAAAGTGGATGAAGATCCAACCCAATCTCTTGAATTGGGTTGGATCTTCATCCACC	Human
shNC	GATCCGTTCTCCGAACGTGTCACGTAATTCAAGAGATTACGTGACACGTTCGGAGAATTTTTTC	AATTGAAAAAATTCTCCGAACGTGTCACGTAATCTCTTGAATTACGTGACACGTTCGGAGAACG	Rat and Human

Table S3. Transcription factors binding to *Gpx4* promoter identified in CistromeDB toolkit.

**Factor**	**RP_score**	**Factor**	**RP_score**
ATF1	1	FOXK2	0.861378
POLR2A	0.965496	MYC	0.856204
USF1	0.954286	EGR1	0.850754
ZNF639	0.937726	RAD21	0.848978
CREB1	0.908264	NFE2	0.840742
USF2	0.88819	RELA	0.835614
RUNX1	0.879902	MED12	0.835494
MAX	0.873586	KLF12	0.827125
FAIRE	0.873499	SPI1	0.820146
BRD4	0.869279	PRKDC	0.809213

Table S4. Proteins identified in shotgun proteomics in ADSCs treated with vehicle or Erastin. (See Table S4.xlsx)
